# Dasatinib exacerbates splenomegaly of mice inoculated with Epstein-Barr virus-infected lymphoblastoid cell lines

**DOI:** 10.1038/s41598-020-61300-y

**Published:** 2020-03-09

**Authors:** Ryutaro Kotaki, Masaharu Kawashima, Yuichiro Yamamoto, Hiroshi Higuchi, Etsuko Nagashima, Natsumi Kurosaki, Masako Takamatsu, Yara Yukie Kikuti, Ken-Ichi Imadome, Naoya Nakamura, Ai Kotani

**Affiliations:** 1 Department of Hematological Malignancy, Institute of Medical Science, Tokai University, Shimokasuya 143, Isehara, Kanagawa Japan; 2 Division of Clinical Oncology and Hematology, The Jikei University School of Medicine, Minato-ku, Tokyo Japan; 3 Research Institute of Science and Technology, Tokai University, 4-1-1 Kitakinme, Hiratsuka, Kanagawa Japan; 4 Department of Pathology, Tokai University School of Medicine, Shimokasuya 143, Isehara, Kanagawa Japan; 5 Department of Infectious Diseases, National Center for Child Health and Development, Setagaya-ku, Tokyo Japan; 6 Precursory Research for Embryonic Science and Technology, Japan Science and Technology Agency, Saitama, Japan; 7 AMED-PRIME, Japan Agency for Medical Research and Development, Tokyo, Japan

**Keywords:** Herpes virus, B-cell lymphoma

## Abstract

Latent infection of Epstein-Barr virus (EBV) is associated with a poor prognosis in patients with B cell malignancy. We examined whether dasatinib, a multi kinase inhibitor, which is broadly used for chronic myeloid leukemia and Philadelphia chromosome-positive acute lymphoblastic leukemia is effective on EBV-positive B cell malignancies, using lymphoblastoid cell lines (LCLs) *in vitro* and *in vivo*. As a result, *in vitro* experiments showed that dasatinib induced cell death of the EBV-LCLs which was not accompanied with a lytic reactivation of EBVs. To evaluate the effectiveness in EBV latency type III represented by immunodeficiency lymphoma, LCL-inoculated immunodeficient NOD/shi-*scid*/*Il2rg*^nul^ (NOG) mice were treated with dasatinib. However, *in vivo* experiments revealed that dasatinib treatment exacerbated tumor cell infiltration into the spleen of LCL-inoculated NOG mice, whereas tumor size at the inoculated site was not affected by the treatment. These results suggest that dasatinib exacerbates the pathogenesis at least in some situations although the drug is effective *in vitro*. Hence, we should carefully examine a possibility of dasatinib repositioning for EBV^+^ B cell malignancies.

## Introduction

Epstein-Barr virus (EBV) is a human oncogenic herpesvirus which persistently infects more than 90% of the healthy adult humans^1,2^. While primary EBV infection in adolescents causes infectious mononucleosis, the infection in childhood is usually asymptomatic. After the primary infection, EBV establishes a lifelong persistent infection preferentially in resting memory B cells^3–5^. Although the persistent EBV infection is usually asymptomatic, EBV may be involved in various B cell malignancies, such as diffuse large B-cell lymphoma (DLBCL), Burkitt lymphoma, classical Hodgkin lymphoma, and post-transplant lymphoproliferative disorder.

Several studies have reported that EBV is associated with a poor prognosis in patients with classical Hodgkin lymphoma and DLBCL, typically in an aged population^6–9^. Malignant EBV^+^ B cells express several genes encoded in the EBV genome, including latent membrane proteins (LMPs) and EBV nuclear antigens (EBNAs)^2^. Based on expression profiles of the EBV-derived molecules, EBV^+^ B cell malignancies are classified into three types of latency, latency I, II, and III. A small-scaled study in our institute suggested that patients with latency III EBV^+^ DLBCL had a very poor prognosis, namely they died within one year from diagnosis, even though they were treated with R-CHOP, a standard therapy for DLBCL^9^. Latency III EBV^+^ cells express most EBV-derived genes among the three types. Hence, the EBV-derived factors may be involved in the poor prognosis of the patients with latency III EBV^+^ DLBCL. One possible therapeutic target for latency III EBV^+^ B cell malignancies is signal transduction pathways from LMPs. LMPs, including LMP1 and LMP2A, which mimic signal transduction from B cell surface molecules, CD40 and B cell antigen receptor, respectively^10^. These LMPs constitutively activate the downstream signal and, hence, enhance survival and proliferation of the infected B cells. *In vitro* EBV infection to human B cells induces immortalization and proliferation of the cells, indicating a transformation activity of EBVs. Such *in vitro* infected B cells, called lymphoblastoid cell lines (LCLs), have been utilized as a useful model for EBV^+^ B cell malignancy. LCLs express EBV-derived genes including LMP1 and LMP2A and are classified as latency III.

Downstream pathways of the EBV LMPs include Src family kinases (SFKs). Previous studies have revealed that LMP1 pathway activates Src, which subsequently activates a transcription factor IRF4^11,12^. B cell-specific LMP1-transgenic mice developed LMP1-positive lymphoma triggered by aging or T cell depletion^13,14^, indicating LMP1 is a sufficient oncogenic factor for EBV^+^ B cell malignancy. Other studies have reported a role of LMP2A in abnormal B cell survival^15,16^. These studies showed early B cell-specific LMP2A-transgenic mice developed B cells without surface immunoglobulin through abnormal B cell selection due to a constitutive survival signal from LMP2A. Further, LMP2A exacerbates lymphomagenesis caused by c-Myc mutation in LMP2A/Myc double transgenic mice^17,18^. In EBV-infected LCLs, c-Myc is highly expressed by EBV-derived transcription factor, EBNA2^19^, suggesting that LMP2A is involved in EBV^+^ B cell malignancy cooperatively with EBNA2. Indeed, LMP2-deficient EBVs showed less efficient transformation of B cells when infected *in vitro*^20^. A survival signal from LMP2A in the transgenic mice requires another SFK, Lyn^21^. Collectively, SFKs, Src and Lyn, are involved in signaling pathways from LMP1 and LMP2A, respectively. Hence, inhibition of SFKs is a possible therapeutic way for EBV^+^ B cell malignancy.

Dasatinib (firstly named BMS-354825) is a multi-target tyrosine-kinase inhibitor, which can inhibit SFKs^22,23^. This orally-available drug has been already approved for use in patients with chronic myeloid leukemia (CML) and Philadelphia chromosome-positive acute lymphoblastic leukemia (Ph^+^ ALL) in US, EU, and Japan. Previous study has reported that dasatinib treatment reduces splenomegaly in LMP2A/Myc double transgenic mice^24^. Furthermore, recent study reported that mTORC2 inhibition in combination with dasatinib could provide a better clinical outcome against DLBCL^25^. Hence, in the present study, we aimed to examine effectiveness of dasatinib in EBV^+^ malignancy, in particular, latency III type represented by immunodeficiency lymphoma^26^. We used EBV-infected LCLs, which express both of LMP1 and LMP2A, and are derived from human B cells. The effects of dasatinib on the LCLs were examined *in vitro* cell culture and *in vivo* LCL-xenograft immunodeficient NOD/shi-*scid*/*Il2rg*^nul^ (NOG) mice.

## Results

### Dasatinib induces cell death and cell cycle arrest of EBV-LCLs *in vitro*

**Figure 1:**
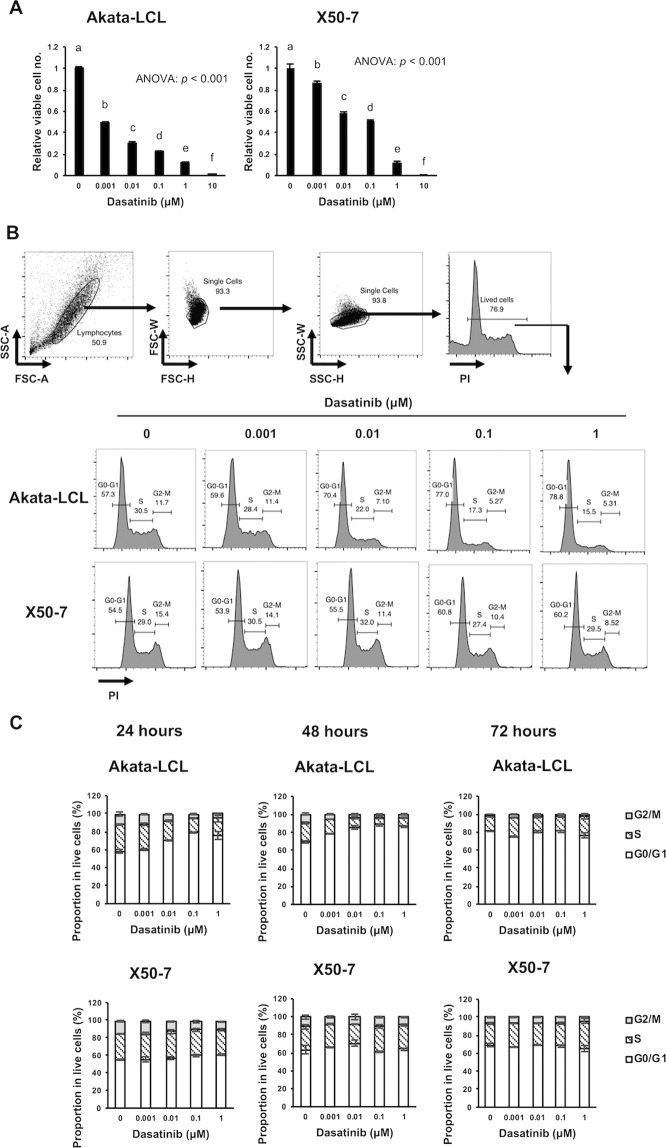
Dasatinib decreases viable cells and induced cell cycle arrest of EBV-LCLs. (**A**) The LCLs were cultured at the density of 2 × 10^4^ cells/mL with indicated doses of dasatinib for 72 hours (*n* = 3), and CellTiter-Glo assay was performed. The relative intensity of luminescence to that of a negative control (0 μM dasatinib) was calculated. (**B,C**) The LCLs were cultured at the density of 1 × 10^5^ cells/mL with indicated doses of dasatinib for 24, 48, and 72 hours (*n* = 3). Subsequently, the cells were fixed with in 70% ethanol with RNase, stained with PI, and analyzed by flow cytometry. (**B**) Gating strategy (upper) and representative plots (bottom) after 24 hours culture are shown. (**C**) Graphs indicates mean ± SD of the proportion of cells in G0/G1, S, G2/M phase after 24, 48, and 72 hours culture. The graphs are representatives of two independent experiments. Statistical analysis was performed using one-way ANOVA and subsequent Tukey’s HSD method. Values not sharing a common letter are significantly different (*p* < 0.05) shown in Fig. 1A and S1.

As models for latency III EBV^+^ B cell malignancy, we used two types of EBV-LCLs, Akata-LCL and X50-7. These LCLs were cultured with dasatinib for 3 days, and the relative number of viable cells was measured by a bioluminescent reaction to ATP from viable cells. Of both LCLs, the viable cell number was decreased by dasatinib treatment in a dose-dependent manner (Fig. 1A). Akata-LCLs seemed to be more sensitive to dasatinib. Next, we checked whether dasatinib treatment affects cell cycle of EBV-LCLs. LCLs were treated with dasatinib for 24, 48, and 72 hours, and proportion of cells in G0/G1, S, and G2/M phases were measured (Figs. 1B,C, and S1). The results showed that dasatinib increased a proportion of G0/G1 phase at 24 and 48 hours in Akata-LCLs, not X50-7 LCLs. These cell cycle arrest may contribute to the decrease in viable cell number after three days treatment shown in Fig. 1A. Such difference in effectiveness on cell cycle of the LCLs is probably due to differences in origins of the host cells, EBV strains, and passage numbers after establishment of the LCLs.

### Cell death induced by dasatinib is mostly non-apoptotic

**Figure 2:**
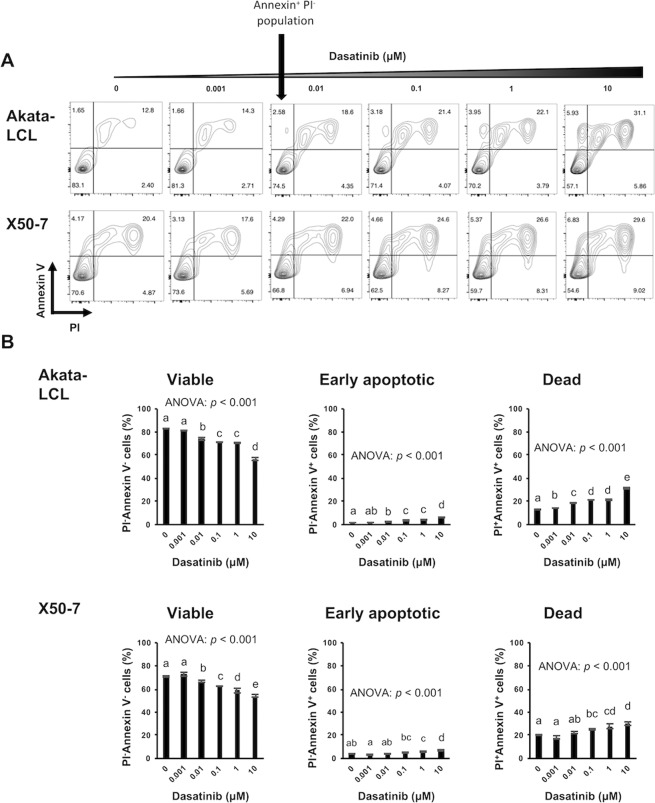
24 hours dasatinib treatment induces mostly non-apoptotic cell death in EBV-LCLs. The LCLs were cultured at the density of 1 × 10^5^ cells/mL with indicated doses of dasatinib for 24 hours (*n* = 3). Subsequently, Annexin V and PI staining of the cells was analyzed by flow cytometry. (**A**) Representative plots are shown. (**B**) The graphs are representatives of two independent experiments (mean ± SD). Statistical analysis was performed using one-way ANOVA and subsequent Tukey’s HSD method. Values not sharing a common letter are significantly different (*p* < 0.05).

**Figure 3:**
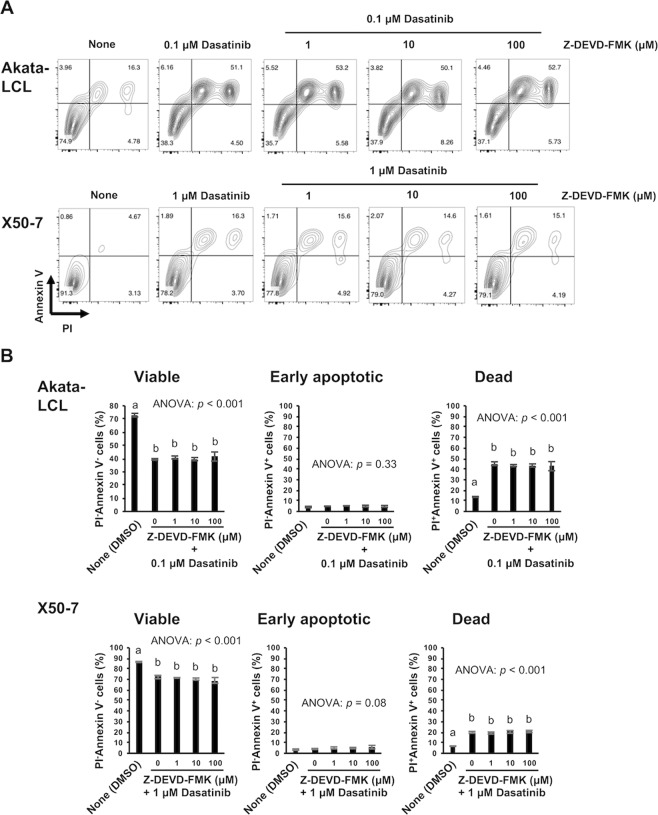
Apoptosis is not induced by dasatinib in EBV-LCLs. The LCLs were cultured at the density of 1 × 10^5^ cells/mL with indicated doses of dasatinib and Z-DEVD-FMK for 72 hours (*n* = 3). Subsequently, Annexin V and PI staining of the cells was analyzed by flow cytometry. (**A**) Representative plots are shown. **(B**) The graphs are representatives of two independent experiments (mean ± SD). Statistical analysis was performed using one-way ANOVA and subsequent Tukey’s HSD method. Values not sharing a common letter are significantly different (*p* < 0.05).

We next check whether dasatinib induces apoptosis. In both LCLs, a proportion of viable cells (Annexin V^−^PI^−^ cells) decreased, and that of dead cells (Annexin V^+^PI^+^ cells) increased in a dose-dependent manner after 24, 48, and 72 hours treatment (Figs. 2 and S2). These results suggest that dasatinib induces cell death of EBV-LCLs resulting in the decrease in viable cell number as described in Fig. 1A. A small proportion of early apoptotic cells (Annexin V^+^PI^−^ cells) was slightly increased in Akata-LCLs by 24 hours dasatinib treatment (Fig. 2). However, Akata-LCLs after 48 and 72 hours and X50-7 at any time point, the apoptotic cell populations were barely observed (Figs. 2, S2 and S3). Further, caspase-3 cleavage, which indicates activation of an apoptotic process, was not detected in the LCLs treated with dasatinib at least after 24 hours (Fig. S4). In addition, a caspase-3 inhibitor, Z-DEVD-FMK, could not suppress the cell death induced by dasatinib (Fig. 3). These results suggest that dasatinib treatment might induce cell death of EBV-LCLs mostly through non-apoptotic process.

### Dasatinib inhibits phosphorylation of SFK and BTK in EBV-LCLs

**Figure 4:**
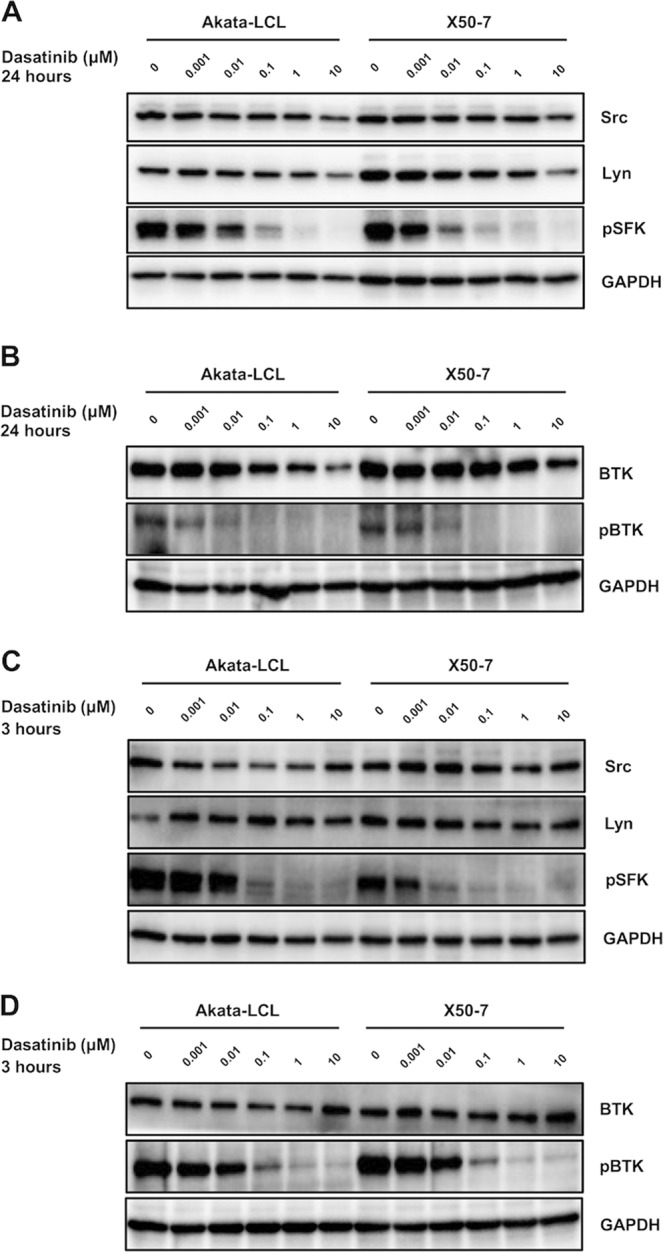
Dasatinib decreases phosphorylation of SFKs and BTK. The LCLs were cultured at the density of 2.5 × 10^5^ cells/mL with indicated doses of dasatinib for (**A,B**) 24 hours or (**C,D**) 3 hours and proteins in lysates of the cells were detected by Western blotting. (**A,C**) Western blot analysis of Lyn, Src and pSFK. (**B,D**) Western blot analysis of BTK and pBTK. The data are representatives of two independent experiments.

We next investigated the effect on SFKs, Src and Lyn by dasatinib treatment, which have been reported to be involved in signaling pathways of EBV-derived molecules^11,12,21^. Dasatinib treatment suppressed phosphorylation of SFKs including Src and Lyn (Fig. 4A), suggesting that the effect of dasatinib could be mediated by inhibition of these signaling pathways.

Knockdown of Src in X50-7 exhibited no change in viable cell number (Fig. S5A,B), showing that partial inhibition of Src is insufficient for suppression of cell proliferation for EBV-LCLs. Furthermore, another SFK inhibitor, saracatinib (AZD0530)^27,28^, could not induce cell death for X50-7 at a dose of 0.1 μM, which was sufficient for loss of phosphorylation of SFK (Fig. S6). Thus, although our result showed that Akata-LCLs are more sensitive to death caused by SFK inhibitor (Figs. 1A, S6A), phosphorylation of SFK in these cells was more resilient after dasatinib treatment when compared to that of X50-7 LCLs (Figs. 4A, S6B), suggesting that other pathways are affected by dasatinib.

Thus, we next investigated whether dasatinib inhibit BTK, which has been reported to activated and partially involved in LMP2A signal pathway^29,30^ and a target of dasatinib in B lymphocytes^31^. Indeed, dasatinib could also inhibit BTK phosphorylation in EBV-LCLs (Fig. 4B). Dasatinib inhibited the phosphorylation of SFKs and BTK at an earlier time point, 3 hours after treatment (Fig. 4C,D). We further examined effects of a BTK inhibitor, ibrutinib (PCI32765)^32,33^, which also inhibits phosphorylation of SFKs at a high dose such as 1 μM^34^. We found that ibrutinib induced cell death of the LCLs only at high dose which was required for inhibiting SFK phosphorylation, but not at low dose which was sufficient for inhibiting BTK phosphorylation (Fig. S7A,B). Collectively, combined inhibition of SFK and BTK phosphorylation, which has been reported to be involved in LMP1 and/or LMP2A signal pathways^11,12,21,29,30^, might partially explain the efficacy of dasatinib in EBV-LCLs, while sole inhibition of SFKs or BTK is insufficient for cell death in EBV-LCLs. In addition, it is probable that other targets of dasatinib are involved in the effects.

### Dasatinib does not induce lytic reactivation of EBVs in LCLs

**Figure 5:**
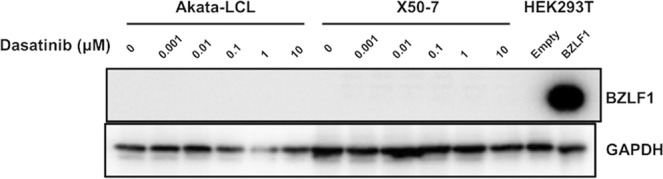
Lytic reactivation of EBV is not induced by dasatinib treatment. The LCLs were cultured at the density of 2.5 × 10^5^ cells/mL with indicated doses of dasatinib for 24 hours, and proteins in lysates of the cells were detected by Western blotting. As controls, lysates from HEK293T cells with or without BZLF1 overexpression were used. The data are representatives of two independent experiments.

In response to certain types of stimuli, latent EBV-infected cells undergo apoptotic cell death accompanied with lytic viral reactivation, which induces viral replication and, hence, should be inhibited for treatment of EBV^+^ tumors. We assessed the induction of an early lytic transcription factor, BZLF1, in LCLs cultured with dasatinib. As a result, BZLF1 was not detected in the LCLs treated with dasatinib (Fig. 5), demonstrating that the dasatinib-induced cell death was not accompanied by lytic reactivation of EBVs. Collectively, dasatinib may induce non-apoptotic cell death of EBV-LCLs without lytic reactivation of EBVs. These results from *in vitro* experiments support a possibility that dasatinib can be used for a treatment of latency III EBV^+^ B cell malignancies.

### Dasatinib does not improve tumorigenesis of LCL-xenograft mice

**Figure 6:**
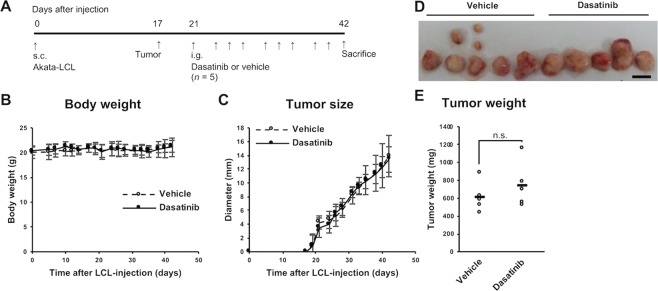
Dasatinib treatment does not affect a tumorigenesis in mice inoculated with EBV-LCLs. NOG mice subcutaneously inoculated Akata-LCLs were treated with dasatinib or vehicle (*n* = 5). (**A**) The experimental schema is described. (**B**,**C**) Body weight and tumor diameter were measured through the experiments. White circles with a dashed line indicate means of vehicle-treated group, whereas black circles with a solid line indicate those of dasatinib-treated group. The error bars show SD. (**D**) The tumors removed at the end of the experiment were photographed. The scale bar indicates 10 mm. (**E**) A total tumor weight at the end of the experiment was analyzed. Statistical analysis was performed using Student’s *t* test (n.s.: not significant (*p* ≥ 0.1)).

Next, we sought to examine whether dasatinib could be used as a therapeutic way in a mouse model. We used an LCL-xenograft mouse model. Akata-LCLs were subcutaneously inoculated into a back of severely immunodeficient NOG mice on day 0. On day 17, we observed tumorigenesis at the inoculated site of several mice (Fig. 6A). After the tumorigenesis was observed in all the mice, we started to administer dasatinib or vehicle by intragastric administration every two or three days (three times a week) for 3 weeks. All the mice survived the experimental procedure and were immediately sacrificed and analyzed on day 42. Body weight and tumor size were not different between the groups treated with the vehicle and dasatinib (Fig. 6B–E). The tumor weight on day 42 was not different between the two groups either (Fig. 6E). These results revealed that dasatinib is not therapeutic at least in this experimental procedure. The tumors were composed of lymphoma cells expressing EBV-encoded small RNA, EBER, in their nuclei, LMP1, and EBNA2 confirming that these tumors were indeed latency III EBV^+^ tumors originated from the LCLs (Figs. S8–S11).

### Dasatinib exacerbates splenomegaly of LCL-xenograft mice

**Figure 7:**
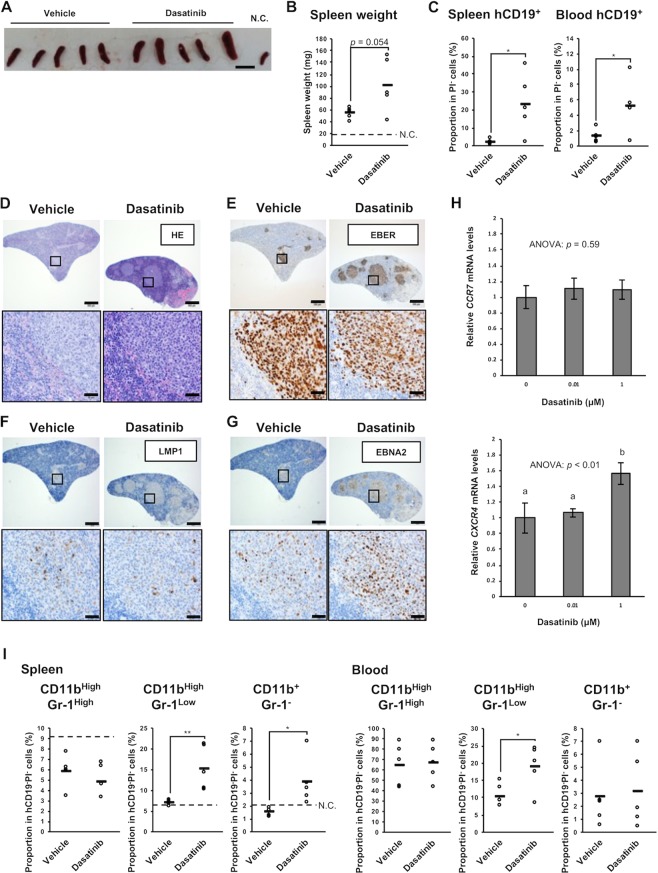
Dasatinib treatment exacerbates splenomegaly of mice inoculated with EBV-LCLs. NOG mice subcutaneously inoculated Akata-LCLs were treated with dasatinib or vehicle (*n* = 5) as shown in Fig. 6A. (**A**) The spleens removed at the end of the experiment were photographed. N.C. (negative control) was the spleen from a non-inoculated NOG mouse. The scale bar indicates 10 mm. (**B**) A spleen weight at the end of the experiment was analyzed. The dashed line shows a weight of the spleen from the non-inoculated NOG mouse. (**C**) A proportion of human CD19^+^ cells in the spleen and peripheral blood at the end of the experiment was analyzed by flow cytometry. Circles indicate data from each mouse, and horizontal bars indicate means. Detailed gating is described in Fig. S12B,C. (**D–G**) The spleen was stained with HE (**D**), EBER-ISH (**E**), anti-LMP1 Ab (**F**), and anti-EBNA2 Ab (**G**). The upper images are of a low-power field (the scale bar indicates 500 μm), whereas the bottom ones are of a high-power field (the scale bar indicates 50 μm). The data are representative of the five mice per group, all of which are shown in Figs. S14–S17. (**H**) Akata-LCLs were treated with dasatinib *in vitro* for 3 hours. Subsequently, CCR7 and CXCR4 mRNA levels were analyzed by qPCR (*n* = 3). Expression levels were normalized to GAPDH. Graphs indicates mean ± SD. Statistical analysis was performed using one-way ANOVA and subsequent Tukey’s HSD method. Values not sharing a common letter are significantly different (*p* < 0.05). (**I**) CD11b and Gr-1 expression on the spleen and peripheral blood cells at the end of the experiment were analyzed by flow cytometry. Circles indicates data from each mouse, and horizontal bars indicate means. The dashed lines show data of the spleen from the non-inoculated NOG mouse. Detailed gating is described in Fig. S18. Statistical analysis was performed using Student’s *t* test (*p < 0.05, **p < 0.01).

We also analyzed the spleen of the LCL-xenograft mice treated with vehicle or dasatinib. Unexpectedly, we found that splenomegaly accompanied by tumorigenesis tended to be exacerbated in the dasatinib-treated mice (Fig. 7A,B). The spleen weight was 53 ± 10 mg (described as mean ± SD) in the vehicle-administered mice and 100 ± 46 mg in the dasatinib-administered ones on day 42 (*p* = 0.054). Thus, we examined infiltration of tumor cells into the spleen. In the spleen and peripheral blood of the dasatinib-treated mice, the inoculated cells, which could be detected as human CD19^+^ cells (Fig. S12A), were increased (Figs. 7C, S12B,C and S13). Further, in the spleen of the dasatinib-treated mice, more EBER^+^ lymphoma cells seemed to have infiltrated compared with the vehicle-treated mice (Figs. 7D,E, S14 and S15). There are clusters of cells that contain some LMP1, but mostly were positive for EBNA2 (Figs. 7F,G, S16 and S17). Taken together, these results suggest that dasatinib treatment increased numbers of EBV^+^ lymphoma cells in the spleen and to exacerbate splenomegaly.

We investigated whether dasatinib treatment up-regulates some chemokine receptors of LCLs and found that gene expression of CXCR4 was increased by dasatinib *in vitro* (Fig. 7H). Human CXCR4 has been reported to be cross-reactive with mouse CXCL12 and to be important for engraftment of human stem cells to NOG mice^35^. Similarly, importance of CXCR4-CXCL12 axis has been reported in engraftment of several human tumor cell lines in xenograft mouse models^36^. Collectively, dasatinib treatment might up-regulate CXCR4 on the LCLs resulting in the enhanced migration of the LCLs into the spleen in the xenograft mice.

In addition, the cell fraction of myelomonocytes were investigated since we reported that monocytes and macrophages support EBV-induced tumorigenesis in another humanized mouse model^37^. We observed an increase in CD11b^High^Gr-1^Low^ cells in the blood and spleen of the dasatinib-treated mice. CD11b^+^Gr-1^−^ monocytes are also increased in the spleen of dasatinib-treated mice (Figs. 7I, S18 and S19). Proportions of these cells correlated well with those of splenic hCD19^+^ cells (Fig. S19). Although only CD11b^High^Gr-1^Low^ markers are insufficient to define, this fraction includes monocyte-like myeloid-derived suppressor cells (MDSCs), which are tumor-induced immunosuppressive cells^38^.

Taken together, although dasatinib induces cell death of EBV-LCLs *in vitro*, the drug exacerbates tumor infiltration into the spleen *in vivo*, at least in the LCL-xenograft mouse model we used. These results suggest that dasatinib may exacerbate the pathogenesis of EBV^+^ B cell malignancies at least in some conditions.

## Discussion

In the present study, we examined effectiveness of dasatinib on EBV-LCLs as a model for latency III EBV^+^ B cell malignancy. We found that dasatinib, which is a multi-target kinase inhibitor and already in clinical use for CML and Ph^+^ ALL, induces cell death in the LCLs *in vitro*. However, single inhibition of pSFK or pBTK are insufficient for inducing cell death. The mechanism for efficacy of dasatinib should be further investigated. In contrast to the *in vitro* experiments, *in vivo* dasatinib administration exacerbated infiltration of LCL-derived latency III tumor cells into the spleen of LCL-xenograft mice.

Previous study has reported that dasatinib treatment reduces tumor growth in LMP2A/c-Myc double transgenic mice probably through inhibiting Lyn phosphorylation^24^. However, in the present study, we found that dasatinib treatment exacerbates tumor infiltration into the spleen of LCL-inoculated mice. These two studies have several differences in mouse models and treatment protocols, and these differences may cause such a discrepancy. The previous study used immunocompetent transgenic mice in which murine B cells express LMP2A and c-Myc transgenes^24^, while we inoculated EBV-LCLs in immunodeficient NOG mice. EBV-LCLs are human B cells transformed by EBV infection and express both of LMP1 and LMP2A, and other EBV-encoded molecules. Hence, EBV-LCLs may have closer properties to those of tumor cells in the human patients. The immunocompetent transgenic mice used in the previous study possess T cells, which can protect against EBV-related lymphomas and may affect influence outcomes of treatment with dasatinib, while immunodeficient NOG mice we used do not possess T cells. Since latency type III EBV^+^ lymphomas occurred mainly in immunocompromised individuals^26^, it is reasonable to use immunodeficient NOG mice in this study. A second difference between the previous and present studies is in the protocols of dasatinib administration. In the previous study, the authors administered 30 mg/kg dasatinib by intraperitoneal injection once a day for 2 weeks, whereas we administered 20 mg/kg dasatinib by intragastric injection three times a week for 3 weeks. Thus, the administration protocol we used in the present study is milder than the previous study. It is possible that such mild treatment of dasatinib accelerates selection of tumor cells with malignant mutations. Moreover, our result demonstrated that *in vitro* dasatinib treatment induced CXCR4 gene upregulation in LCLs. CXCR4 plays an important role in engraftment of tumors^36^, and other reports showed that CXCR4 expression enhances B cell lymphoma dissemination and worsens patient survival^39^. Thus, dasatinib might promote migration of LCLs through upregulation of CXCR4, and further studies are needed. In addition, we observed an increase in CD11b^High^Gr-1^low^ cells (MDSC-like populations) in the spleen of dasatinib-treated mice. This population may be related to tumor progression since MDSCs have been reported as immunosuppressive functions to promote tumor progress^40,41^. Further, CD11b^+^Gr-1^−^ (monocyte populations) were also increased in the spleen of dasatinib-treated mice. Our group previously found that monocytes and macrophages play a supportive role in tumorigenesis of EBV^+^ lymphoma in a humanized mouse model^37^. Hence, it is also possible that the increase in these cells is a cause of tumor progression in the dasatinib-treated mice. However, it is unclear whether the increase in both MDSC-like and monocyte populations is a cause or a result of the exacerbated tumor infiltration. Collectively, it is plausible that dasatinib treatment can result in both improving and exacerbating disease progression in the patients with EBV^+^ B cell malignancy depending on a dose and a schedule of the treatment. In a clinical use, the proper dose of dasatinib might be different individually. Hence, we should carefully investigate the optimal dose and treatment procedure of dasatinib for application to the patients with EBV^+^ B cell malignancy in the future.

Our *in vitro* experiments revealed that dasatinib caused cell death of the EBV-LCLs. However, this cell death is unlikely to be an apoptotic process, since we observed slightly increase in Annexin V^+^PI^−^ cells fractions only in Akata-LCLs for 24 hours dasatinib treatment, no caspase-3 cleavage, and no inhibition of the cell death by a caspase-3 inhibitor. There are several non-apoptotic programmed cell deaths, such as necroptosis and pyroptosis^42–44^. It is possible that dasatinib induces these non-apoptotic cell deaths in the LCLs. In that case, we should note that these cell deaths accompanied by inflammatory responses, such as secretion of IL-1β, IL-18, and cytosolic damage-associated molecular patterns. These inflammatory responses might impair the effects of dasatinib in the patients with EBV^+^ B cell malignancy when treated. On the other hand, we confirmed that dasatinib does not induce lytic reactivation of EBVs, supporting a possibility of dasatinib use for EBV^+^ tumors. This is consistent with a previous study which has reported that dasatinib suppresses lytic reactivation of EBV *in vitro* induced by anti-IgG stimulation^45^.

Taken together, by *in vitro* experiments, we revealed that dasatinib treatment is possibly effective for latency type III EBV^+^ B cell malignancies. Meanwhile, by *in vivo* experiments, we also found that dasatinib treatment, in a mild administration protocol, may exacerbate progression of the tumors. Hence, we should carefully explore the possibility of dasatinib application for treatment of the patients with EBV^+^ B cell malignancies.

## Materials and Methods

### Cells

Akata-LCL was established from human umbilical cord blood B cells infected with Akata strain of EBVs. X50-7, established from human umbilical cord blood lymphocytes infected by B95-8 strain of EBVs^46^, was kindly provided. All cell lines were cultured in RPMI1640 medium (Wako) supplemented with 10% fetal bovine serum (Hyclone), 1% sodium pyruvate (Wako), 1% penicillin and streptomycin (Wako), 1% non-essential amino acids (Wako), and 50 μM 2-mercaptoethanol (Gibco). The cells were incubated in 37 °C humid atmospheres, and 1/4–1/3 volume of the cells were passaged twice a week.

### Reagents

Dimethyl-sulfoxide (DMSO) was purchased from Wako, Japan. Dasatinib (Sigma), saracatinib (Selleck), ibrutinib (Medchemexpress), Z-DEVD-FMK (Selleck), and dexamethasone (Sigma) were dissolved in the DMSO and were stored at −80 or −20 °C.

### Viable cell number measurement

Viable cell number was assayed using CellTiter Glo (Promega). Briefly, 1 × 10^4^ cells were cultured in 48 well plates for 3 days. After the plates were put at a room temperature for 30 minutes, 1/10 of the cultured cells were mixed with equivalent aliquot of CellTiter Glo reagent in 96 well plates and were put at a room temperature for 10 minutes. Subsequently, luminescence was measured using GloMax-Multi Detection System (Promega).

### Flow cytometry

Cells were stained with fluorescence-labeled antibodies and propidium iodide (PI) diluted in 1× annexin binding buffer (BD Biosciences) or PBS containing 2% FBS. Flow cytometry was performed using FACSVerse (BD Biosciences). Data were analyzed using FlowJo software (Tree Star). For flow cytometry, subsequent antibodies and reagents were used: APC-conjugated anti-human CD19 (HIB19, BioLegend), FITC-conjugated anti-mouse Gr-1 (RB6-8C5, BD Pharmingen), PE/Cy7-conjugated anti-mouse/human CD11b (M1/70, BioLegend), APC-conjugated Annexin V (BD Biosciences), and PI (Sigma) dissolved in PBS.

### Western blotting

Cells were suspended in RIPA buffer (Wako) with phosphatase inhibitor cocktail (Nacalai Tesque) and Protease Inhibitor Cocktail for use with mammalian cell and tissue extracts (Sigma) and were put for 15 minutes on ice. The suspensions were centrifuged at 4 °C and a speed of 25000 rpm for 10 minutes, and supernatants were mixed with 4× sample buffer and 5% v/v 2-mercaptoethanol and boiled at 95 °C for 5 minutes. Subsequently, SDS-PAGE was performed with 10–12% polyacrylamide gels. Proteins in the gels were transferred to PVDF membranes through semidry blotting using HorizeBLOT 4M-R (Atto). The membranes were washed with TBS-Tween and were shaken in Can Get Signal PVDF Blocking Reagent (Toyobo) at a room temperature for 1 hour. After washed, the membranes were incubated with primary antibodies diluted in Can Get Signal Solution 1 (Toyobo) at a room temperature for 1 hour or at 4 °C overnight. After washed, the membrane was incubated with secondary antibodies conjugated to horseradish peroxidase (HRP) diluted in Can Get Signal Solution 2 (Toyobo) at a room temperature for 1 hour. After washed, Immobilon (Merck) was added, and luminescence was detected using ChemiDoc Touch Imaging System (Biorad).

For western blotting, subsequent antibodies were used: anti-Src (32G6, rabbit monoclonal antibody (mAb), Cell Signaling Technology), anti-Lyn (C13F9, rabbit mAb, Cell Signaling Technology), anti-pSFK (Tyr416) (rabbit polyclonal Ab, Cell Signaling Technology), anti-GAPDH (rabbit polyclonal Ab, Sigma), anti-BZLF1 (BZ-1, mouse mAb, Santa Cruz Biotechnology), anti-Cleaved Caspase-3 (5A1E, rabbit mAb, Cell Signaling Technology), anti-BTK (D3H5, rabbit mAb, Cell Signaling Technology), anti-human pBTK (Y223) (rabbit polyclonal Ab, Cell Signaling Technology), Rabbit IgG HRP Linked F(ab’)2 Fragment (Amersham Biosciences), and Rabbit IgG HRP Linked F(ab’)2 Fragment (Amersham Biosciences).

### Knockdown of Src by lipid-mediated transfection

Lipofectamine 3000 (Thermo Fisher Scientific) was used for knockdown of control siRNA or Src SiRNA in X50-7 according to manufacturer’s protocol. The human Src siRNA (Target sequence: 5′-UGUUCGGAGGCUUCAACUCCU-3′) and AccuTarget Negative control siRNA (Bioneer) were prepared. Block-iT^TM^ Fluorescent Oligo (Invitrogen) was also prepared and co-transfected with each siRNA to detect transfected fractions (FITC^+^ populations) by FACS analysis.

### *In vivo* administration of dasatinib

Female NOG mice were obtained from the Central Institute for Experimental Animals, Japan. Akata-LCLs (4 × 10^6^ cells/100 μL PBS) were subcutaneously injected on day 0. From day 21, dasatinib or DMSO diluted in 80 mM citrate buffer (pH 3.1) were administrated to the mice by oral gavage (20 mg/kg, 100 μL) every two or three days. The mice were reared in a specific-pathogen-free condition and were treated in accordance with the institutional guidelines for animal care and treatment in experimental investigations.

### Histology, *in situ* hybridization, and immunohistochemistry

Tissues from mice were fixed in 20% HCHO with being shaken gently at a room temperature overnight. The fixed tissues were embedded into paraffin blocks and were cut into thin section on slide glasses. These processes and subsequent hematoxylin eosin staining were performed by Dept. of Cell Biology and Histology, Support Center for Medical Research and Education, Tokai University. *In situ* hybridization and immunohistochemistry staining were performed as previously reported^47^, using Bond ready-to-use ISH EBER probe (Leica Microsystems), anti-LMP1 (CS-1, CS-2, CS-3, and CS-4, mouse mAbs, Leica biosystems), and anti-EBNA2 (PE2, mouse mAb, Novus Biologicals). The tissue slides were observed and were photographed using Olympus BX63 microscope and cellSence software.

### qPCR

Akata-LCLs were treated by dasatinib for 3 hours and purified using Sepasol-RNA I Super G. RT-PCR was conducted with the High-Capacity Reverse Transcription Kit (Applied Biosystems), and quantification by real-time PCR was performed using the THUNDERBIRD SYBR qPCR Mix (TOYOBO CO., Life Science Department Osaka, Japan) following manufacturer’s protocol. The primers were as follows.

humanCCR7: 5′-CAACATCACCAGTAGCACCTGTG-3′ (Forward).

5′-TGCGGAACTTGACGCCGATGAA-3′ (Reverse).

humanCXCR4: 5′-CTCCTCTTTGTCATCACGCTTCC-3′ (Forward).

5′-GGATGAGGACACTGCTGTAGAG-3′ (Reverse).

humanGAPDH: 5′-CTGCACCACCAACTGCTTAG-3′ (Forward).

5′-GCTTCTTTAGATGTCATATCAGCTCA-3′ (Reverse).

### Study approval

All animal procedures and protocols were reviewed and approved by the Animal Care Committee of Tokai University, and all experiments were performed in accordance with the relevant guidelines and regulations.

## Supplementary information

Supplemental Information.
