# Supplementary material for: Dasatinib exacerbates splenomegaly of mice inoculated with Epstein-Barr virus-infected lymphoblastoid cell lines
Source: Sci Rep. 2020 Mar 9;10:4355. doi: 10.1038/s41598-020-61300-y (PMC7062761; doi:10.1038/s41598-020-61300-y)
Supplement: Supplementary file 1 — Supplemental Information. [file 41598_2020_61300_MOESM1_ESM.pdf]

Supplementary Information for

Dasatinib exacerbates splenomegaly of mice inoculated with Epstein-Barr virus-infected lymphoblastoid cell lines.

Ryutaro Kotaki[1)], Masaharu Kawashima[1, 2)], Yuichiro Yamamoto[1)], Hiroshi Higuchi[1, 3)], Etsuko Nagashima[1)], Natsumi Kurosaki[1)], Masako Takamatsu[1)], Yara Yukie Kikuti[4)], Ken-Ichi Imadome[5)], Naoya Nakamura[4)], and Ai Kotani[1, 6, 7)]

[1]Department of Hematological Malignancy, Institute of Medical Science , Tokai University, Shimokasuya 143, Kanagawa, Japan

[2]Division of Clinical Oncology and Hematology, Jikei University School of Medicine, Minato-ku, Tokyo, Japan

[3]Research Institute of Science and Technology, Tokai University, 4-1-1 Kitakinme, Hiratsuka, Kanagawa, Japan

[4]Department of Pathology, Tokai University School of Medicine, Shimokasuya 143, Isehara, Kanagawa, Japan

[5]Department of Infectious Disease, National Center for Child Health and Development, Setagaya-ku, Tokyo, Japan

[6]Precursory Research for Embryonic Science and Technology, Japan Science and Technology Agency, Saitama, Japan

[7]AMED-PRIME, Japan Agency for Medical Research and Development, Tokyo, Japan

Supplementary Figures S1 to S19

Figure S1.

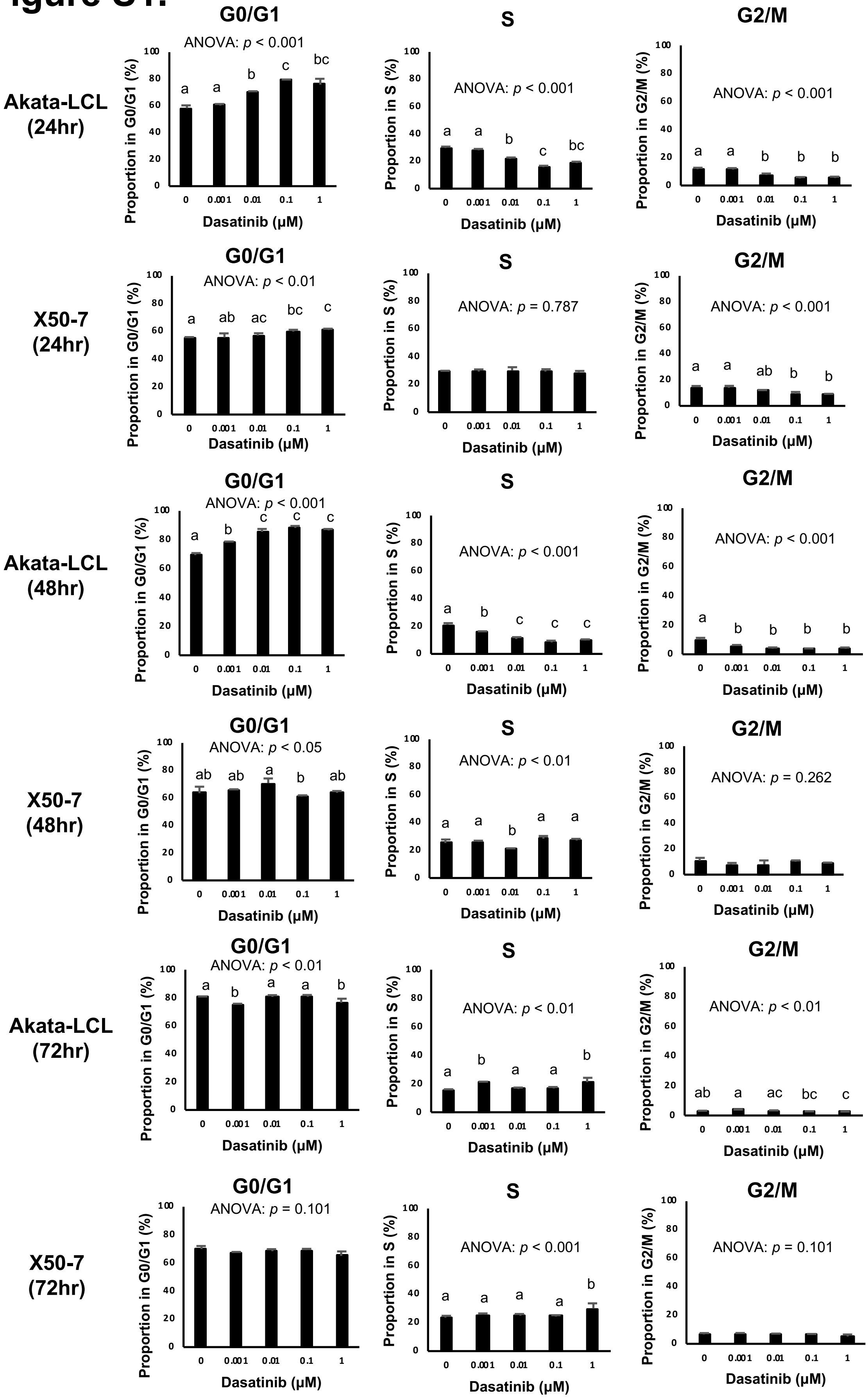

Figure. S1. Dasatinib induces cell cycle arrest of EBV-LCLs *in vitro*.
The graphs of each phases of cell cycle (Fig. 1) are shown. Error bar: mean ± SD. Statistical analysis was performed using one-way ANOVA and subsequent Tukey's HSD method. Values not sharing a common letter are significantly different. ($p < 0.05$).

Figure S2.

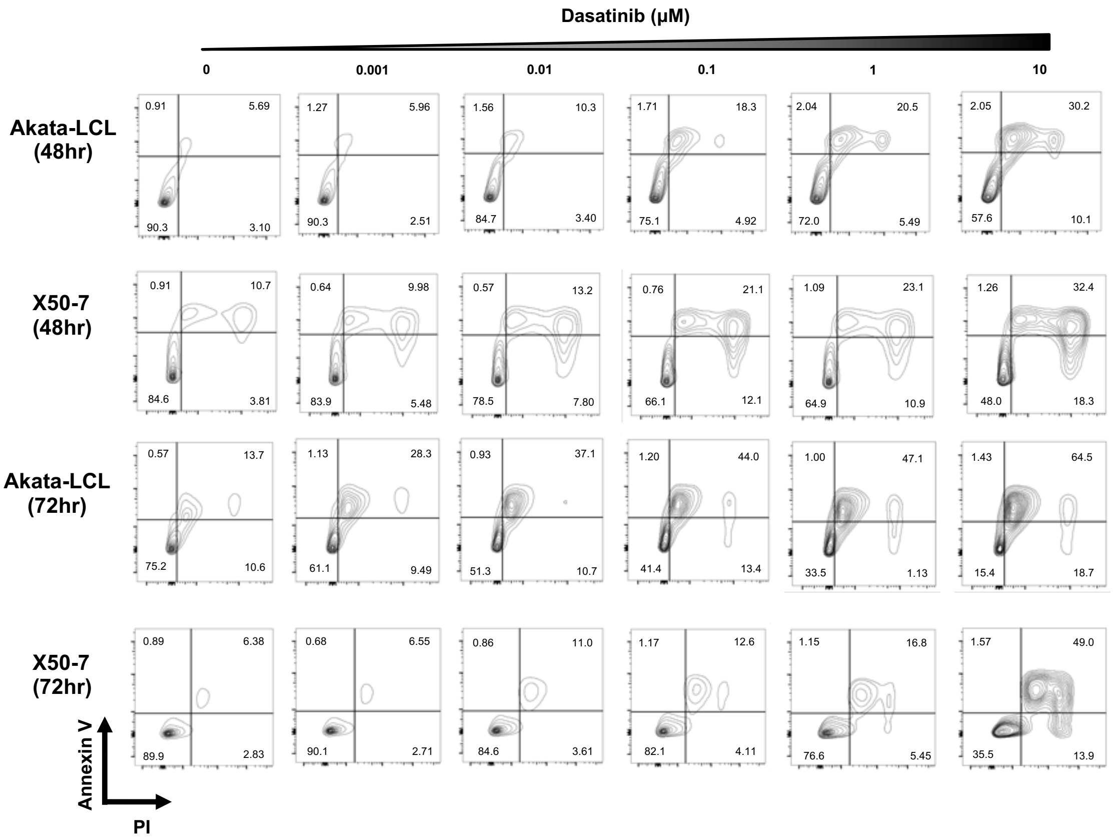

Figure S2. Annexin V$^+$PI$^-$ populations were barely observed after 48 and 72 hours dasatinib treatment. The LCLs were cultured at the density of 1×10^5 cells/mL with indicated doses of dasatinib for 48 and 72 hours (n=3). Subsequently, Annexin V and PI staining of the cells were analyzed by flow cytometry. Representative plots are shown .

Figure S3.

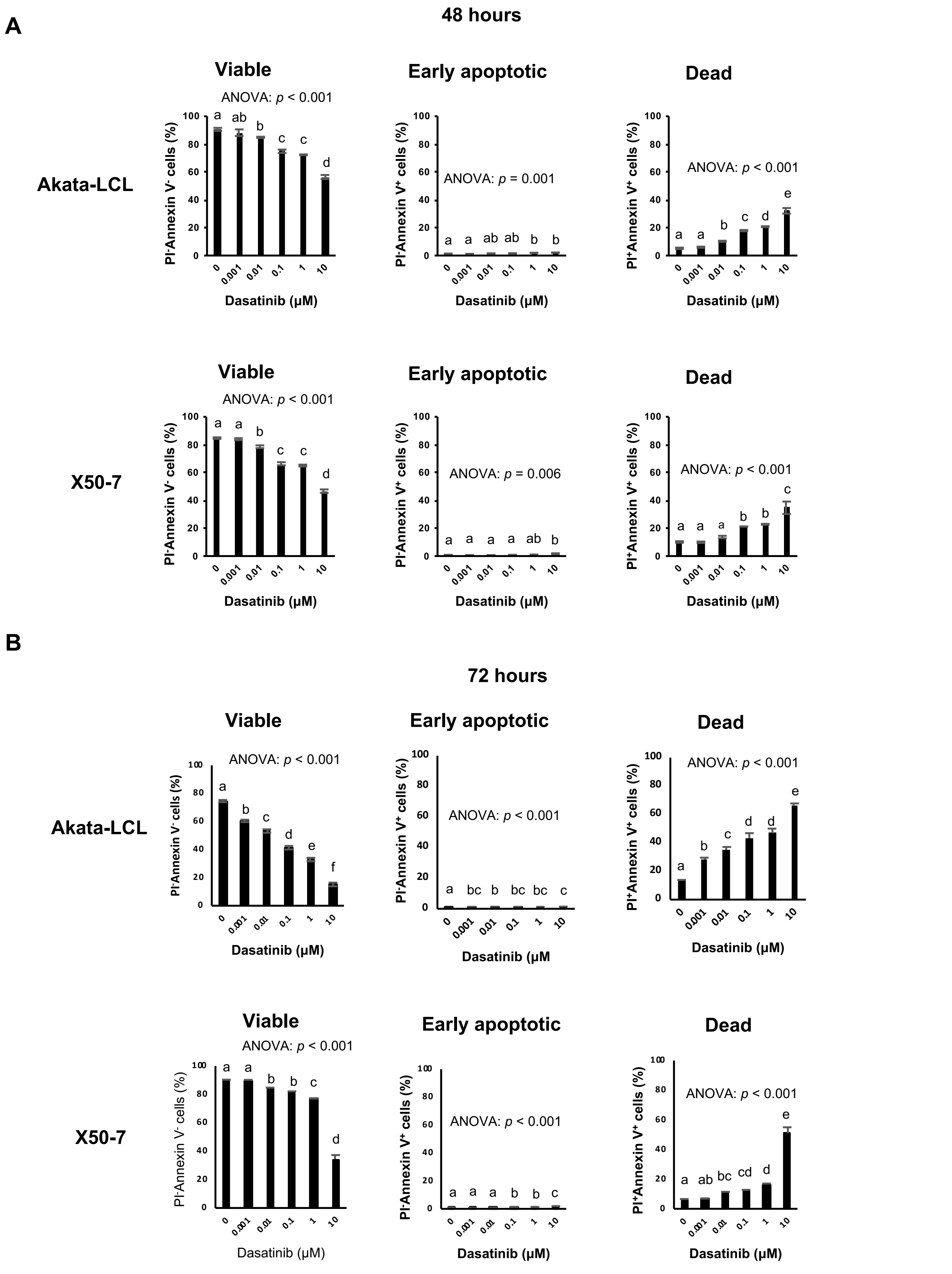

Figure. S3 48 and 72 hour dasatinib treatment induce mostly no apoptotic cell death in EBV-LCLs.
The LCLs were cultured at the density of 1 × 10^5 cells/mL with indicated doses of dasatinib for 48 and 72 hours (n=3). Subsequently, Annexin V and PI staining of the cells were analyzed by flow cytometry. Error bar: mean ± SD. Statistical analysis was performed using one-way ANOVA and subsequent Tukey's HSD method.
Values not sharing a common letter are significantly different. ($p < 0.05$).

Figure S4.

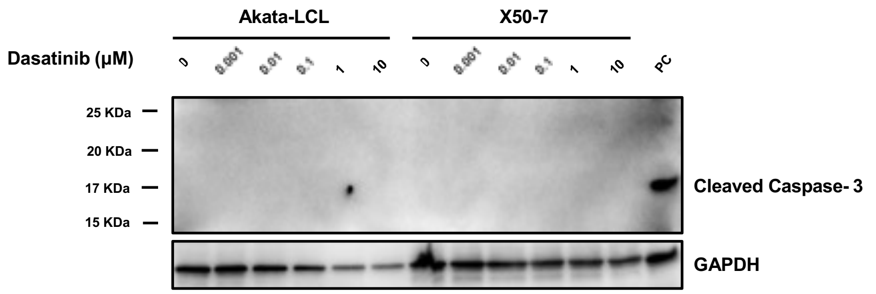

Figure S4. Caspase-3 cleavage is not induced by dasatinib in EBV-LCLs.
The LCLs were cultured at the density of 2.5×10^5 cells/mL with indicated doses of dasatinib for 24 hours, and proteins in lysates of the cells were detected by Western blotting. As Positive Control (PC), lysates from thymocytes of C57BL/6 mice (16 weeks of age) treated with 10 μM dexamethasone for 6 hours were used. The data are representatives of two independent experiments.

Figure S5.

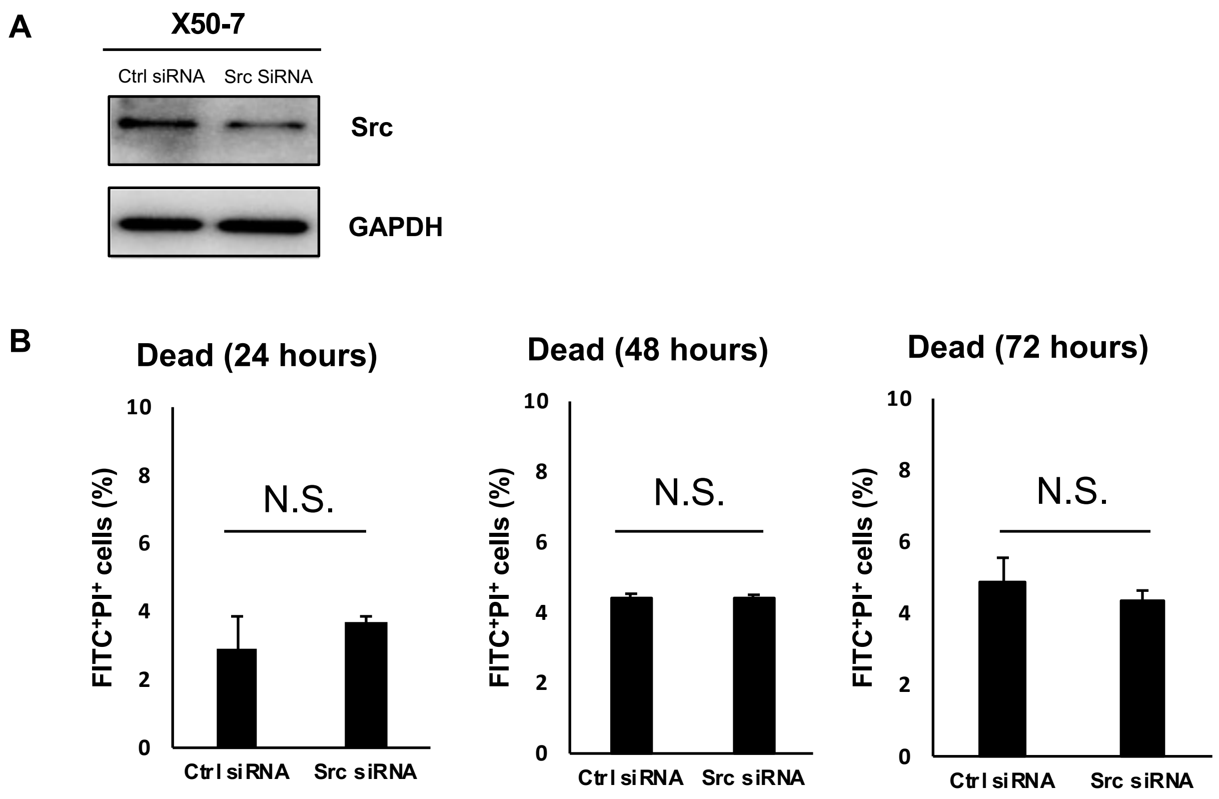

Figure S5. Knockdown of Src is insufficient for suppressing cell death for EBV-LCLs.
(A) X50-7 were transfected with control siRNA or Src SiRNA for 48 hours, and transfected cells were sorted. knockdown of Src was observed by Western blotting. The data are representatives of two independent experiments. (B) X50-7 were transfected with control siRNA or Src SiRNA for 24, 48, and 72 hours, and dead cells were stained with PI. The percentage of transfected dead ($FITC^{+}PI^{+}$) cells were analyzed by FACS at each time course (n = 3). Error bar: mean ± SD. N.S.: not significant. The data were analyzed by Student’s t test.

Figure S6.

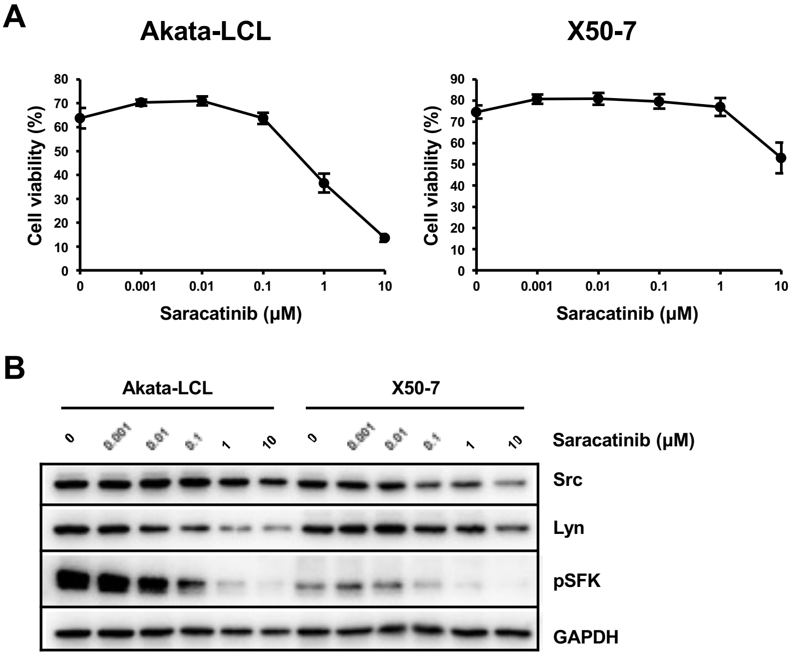

Figure S6. High dose of saracatinib is required to induce cell death in EBV-LCLs .
(A) The LCLs were cultured at the density of 1×10^5 cells/mL with indicated doses of saracatinib for 72 hours. The cells were stained with Annexin V -APC and PI and were analyzed by flow cytometry. Mean ± SD of Annexin V^-PI^- cell proportion was analyzed.
(B) The LCLs were cultured at the density of 2.5×10^5 cells/mL with indicated doses of saracatinib for 24 hours. Lysates from the cells were analyzed by Western blotting.

Figure S7.

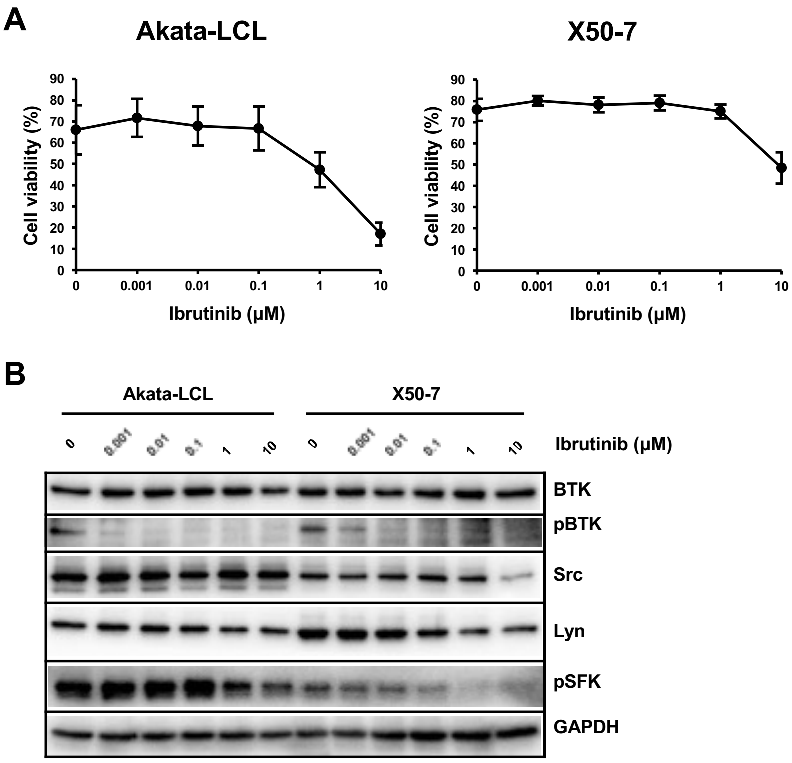

Figure S7. Only BTK phosphorylation inhibition is difficult to induce cell death in EBV-LCLs.
(A) The LCLs were cultured at the density of 1×10^5 cells/mL with indicated doses of ibrutinib for 72 hours. The cells were stained with Annexin V -APC and PI and were analyzed by flow cytometry. Mean ±SD of Annexin V^-PI^- cell proportion was analyzed.
(B) The LCLs were cultured at the density of 2.5×10^5 cells/mL with indicated doses of ibrutinib for 24 hours. Lysates from the cells were analyzed by Western blotting.

Figure S8.

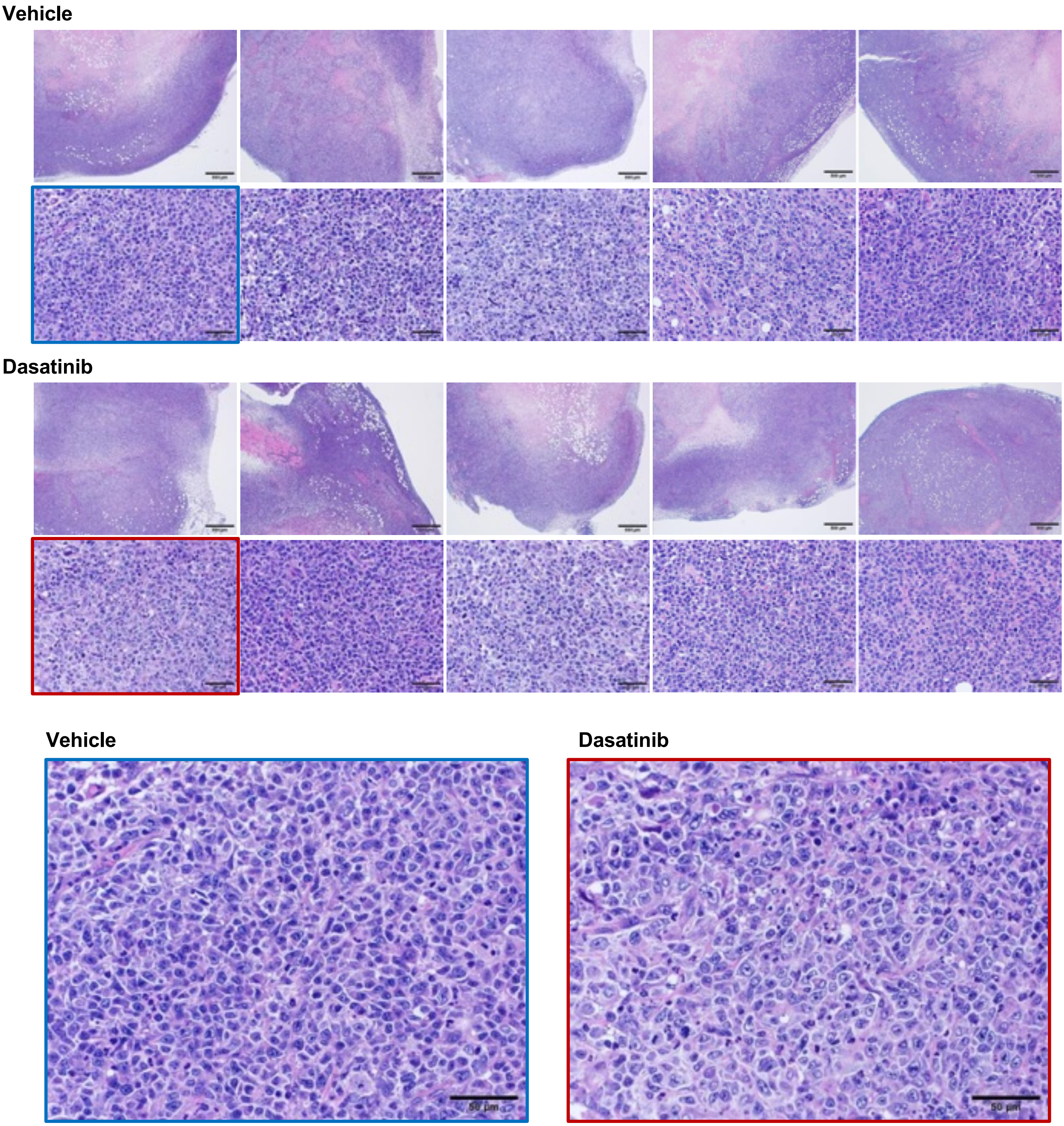

Figure S8. Tumorigenesis is not affected by dasatinib treatment. (1)
Tissue sections of tumors stained with hematoxylin and eosin were analyzed. The small images are from all of the mice treated with the vehicle or dasatinib (*n* = 5). Upper is low-powered images (scale bars: 500 μm), whereas lower is high-powered ones (scale bars: 50 μm), in each sample. On the bottom, representative high-powered images of vehicle- (blue-framed) and dasatinib- (red-framed) treated mice are enlarged for visibility.

Figure S9.

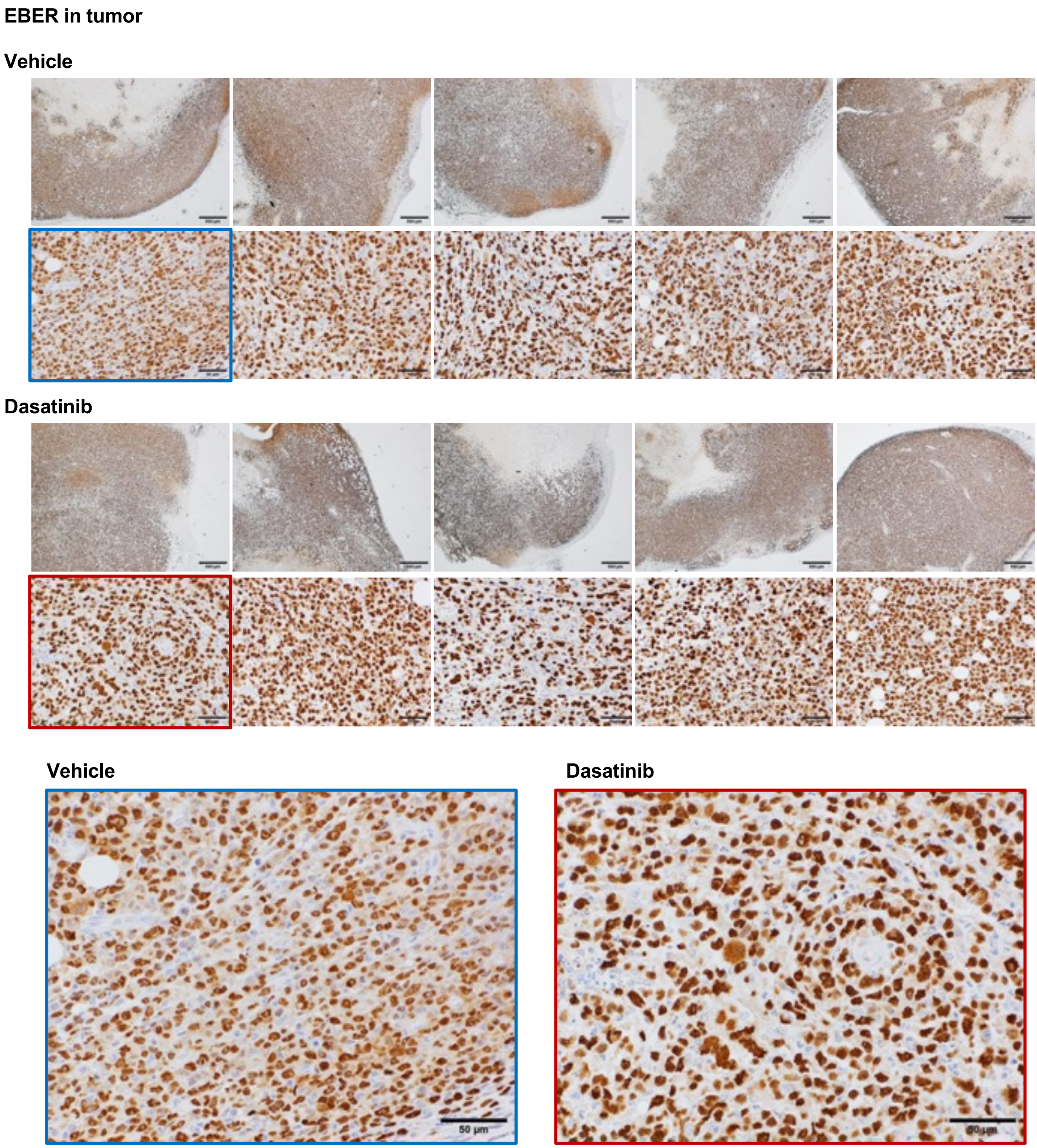

Figure S9. Tumorigenesis is not affected by dasatinib treatment. (2)
Tissue sections of tumors stained with EBER ISH were analyzed. The small images are from all of the mice treated with the vehicle or dasatinib (n = 5). Upper is low-powered images (scale bars: 500 µm), whereas lower is high-powered ones (scale bars: 50 µm), in each sample. On the bottom, representative high-powered images of vehicle- (blue-framed) and dasatinib- (red-framed) treated mice are enlarged for visibility.

Figure S10.

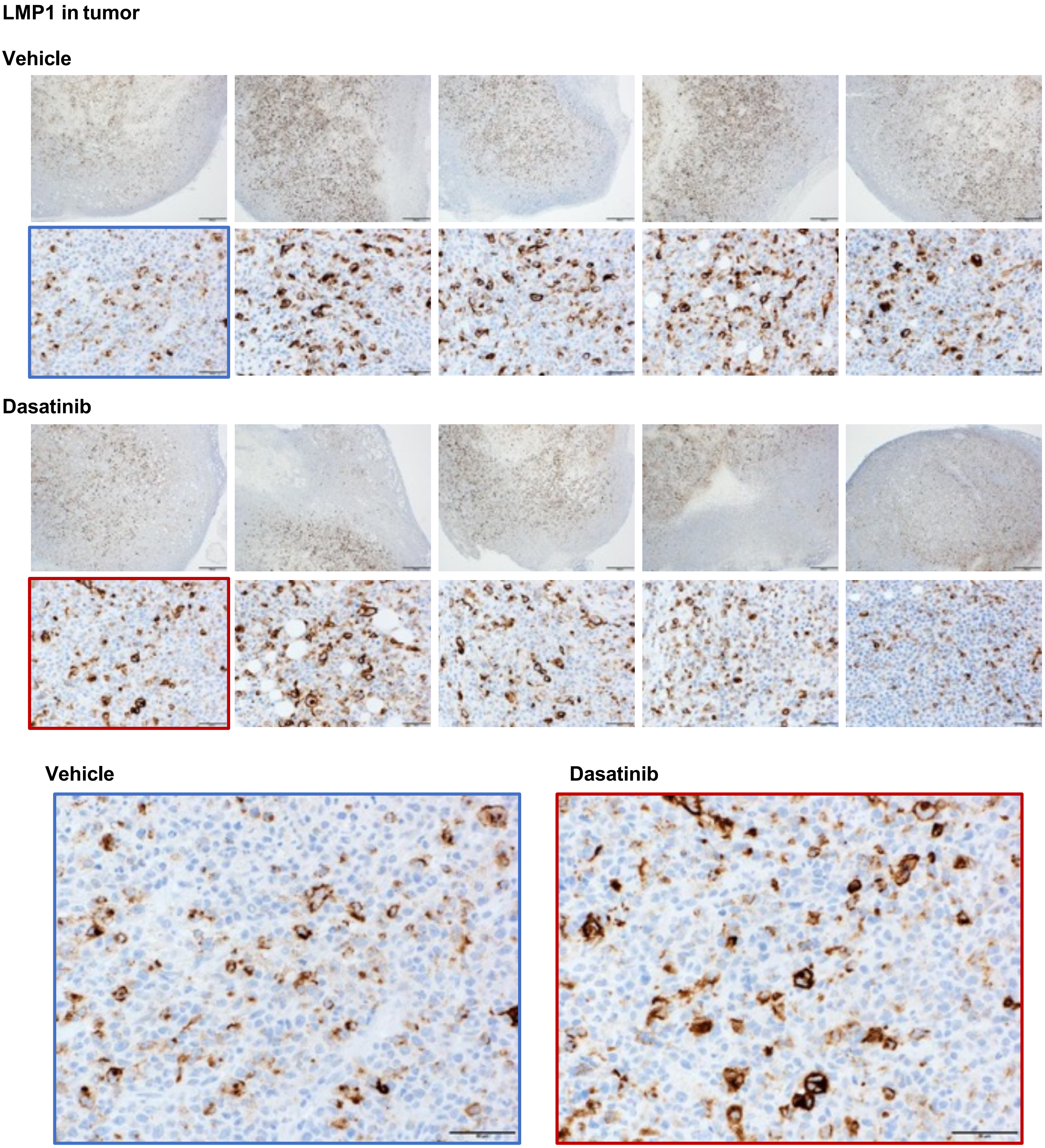

Figure S10. Tumorigenesis is not affected by dasatinib treatment. (3)
Tissue sections of tumors stained with anti-LMP1 Ab were analyzed. The small images are from all of the mice treated with the vehicle or dasatinib (*n* = 5). Upper is low-powered images (scale bars: 500 µm), whereas lower is high-powered ones (scale bars: 50 µm), in each sample. On the bottom, representative high-powered images of vehicle- (blue-framed) and dasatinib- (red-framed) treated mice are enlarged for visibility.

Figure S11.

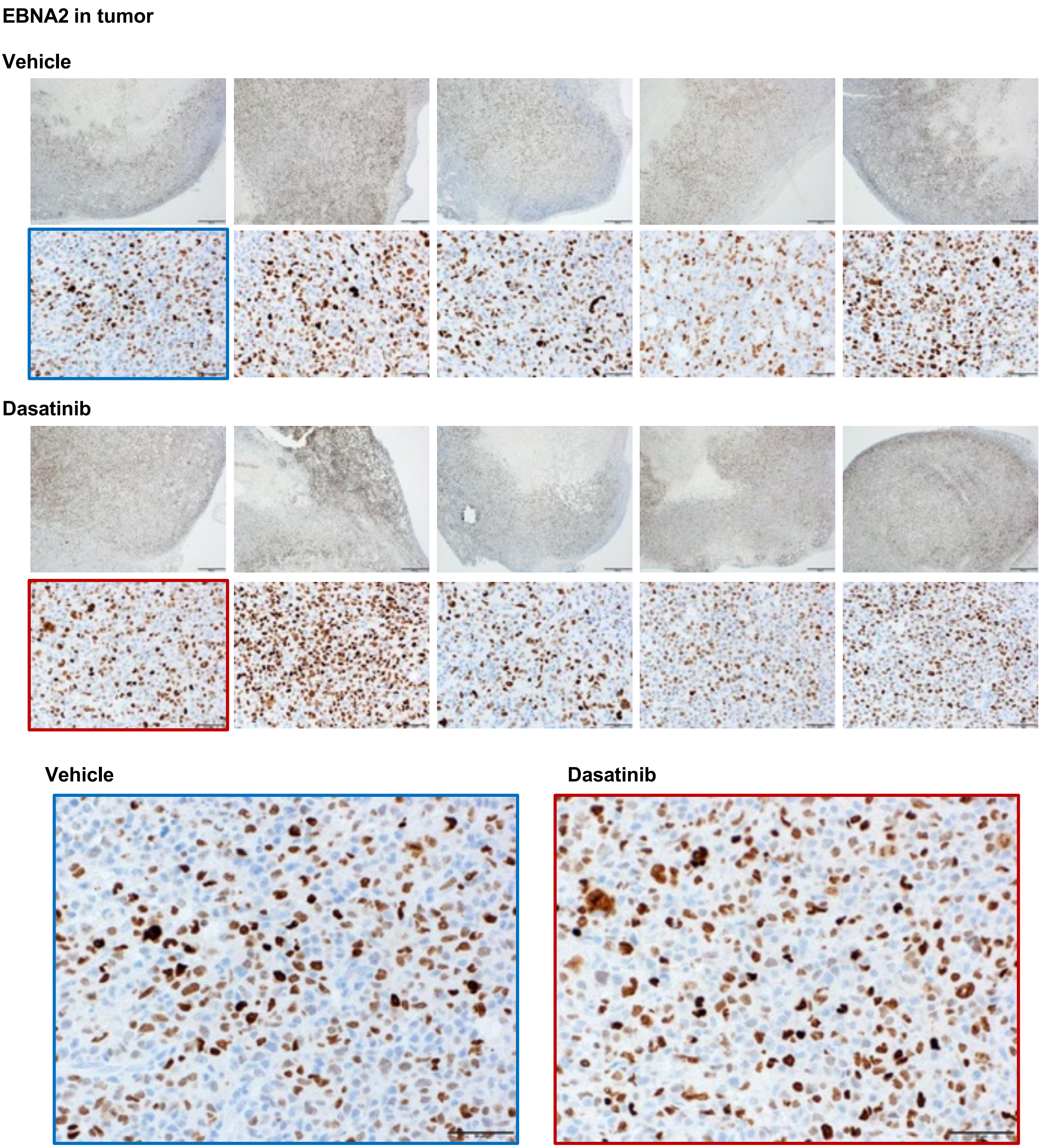

Figure S11. Tumorigenesis is not affected by dasatinib treatment. (4)
Tissue sections of tumors stained with anti-EBNA2 Ab were analyzed. The small images are from all of the mice treated with the vehicle or dasatinib (*n* = 5). Upper is low-powered images (scale bars: 500 μm), whereas lower is high-powered ones (scale bars: 50 μm), in each sample. On the bottom, representative high-powered images of vehicle- (blue-framed) and dasatinib- (red-framed) treated mice are enlarged for visibility.

Figure S12.

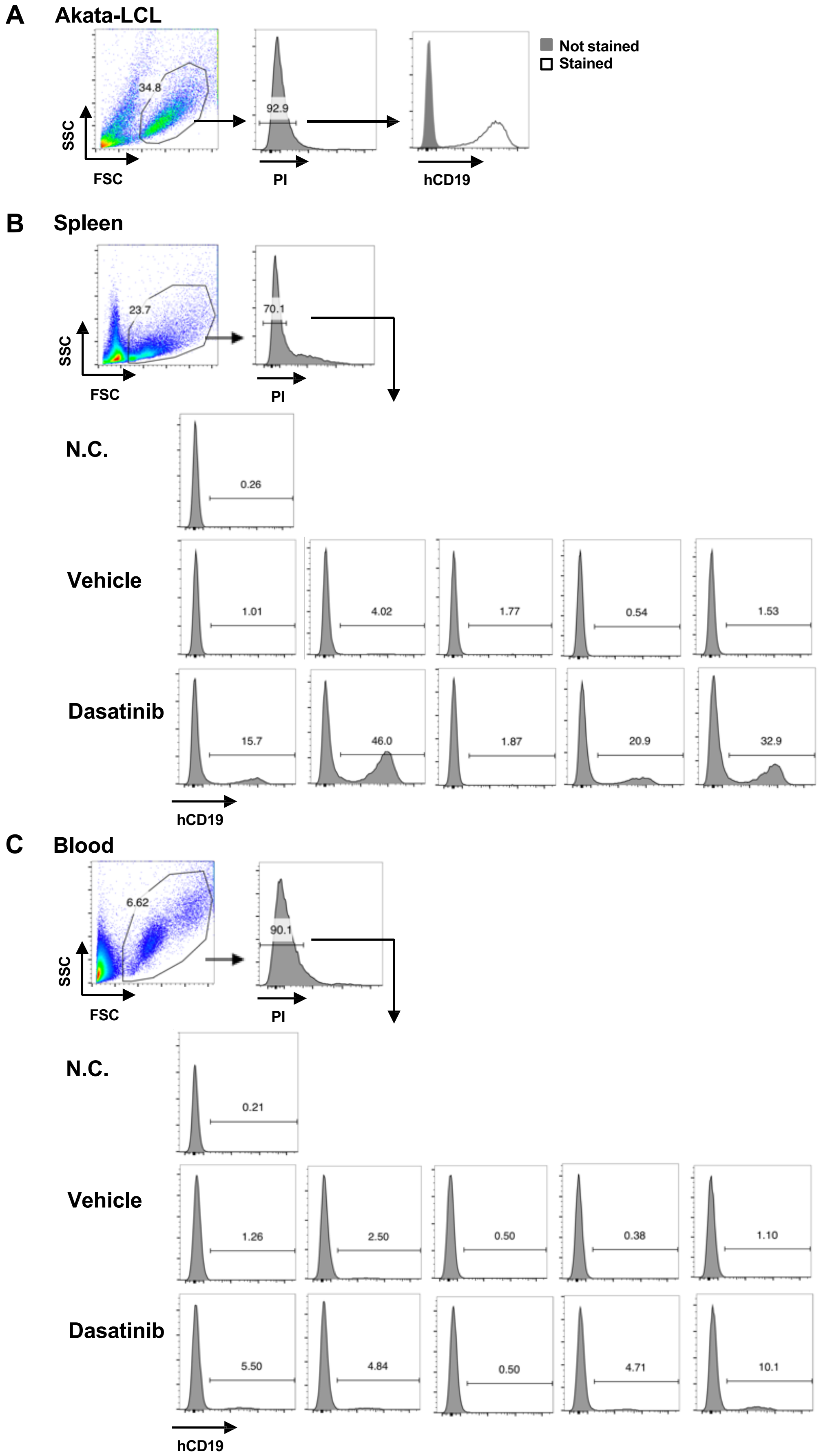

Figure S12. Dasatinib treatment increases hCD19⁺ cells in the spleen and peripheral blood.
Cells were analyzed by flow cytometry.
(A) hCD19 expression on live (PI⁻) Akata-LCLs were analyzed.
(B and C) hCD19 expression on live (PI⁻) cells in the spleen (B) and the peripheral blood (C) of the vehicle- or dasatinib-treated mice were analyzed (*n* = 5). N.C. (negative control) indicates NOG mouse without tumor inoculation.

Figure S13.

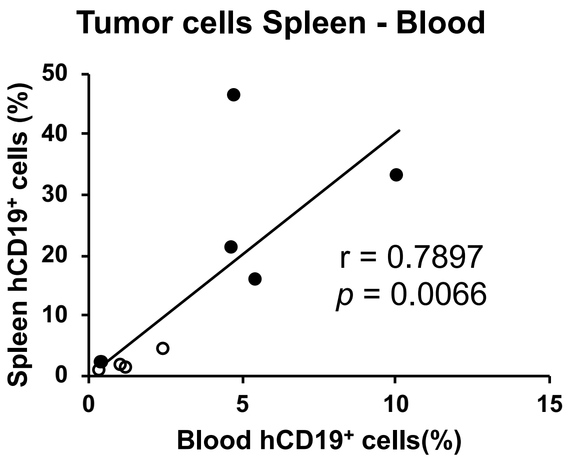

Figure S13. Tumor cells of spleen – blood showed positive correlation.
Correlation of indicated cells in spleen and blood of the vehicle (white plot) or dasatinib-treated (black plot) mice were analyzed (n = 5) using Pearson correlation. r (correlation coefficient) and p values are shown in figure.

Figure S14.

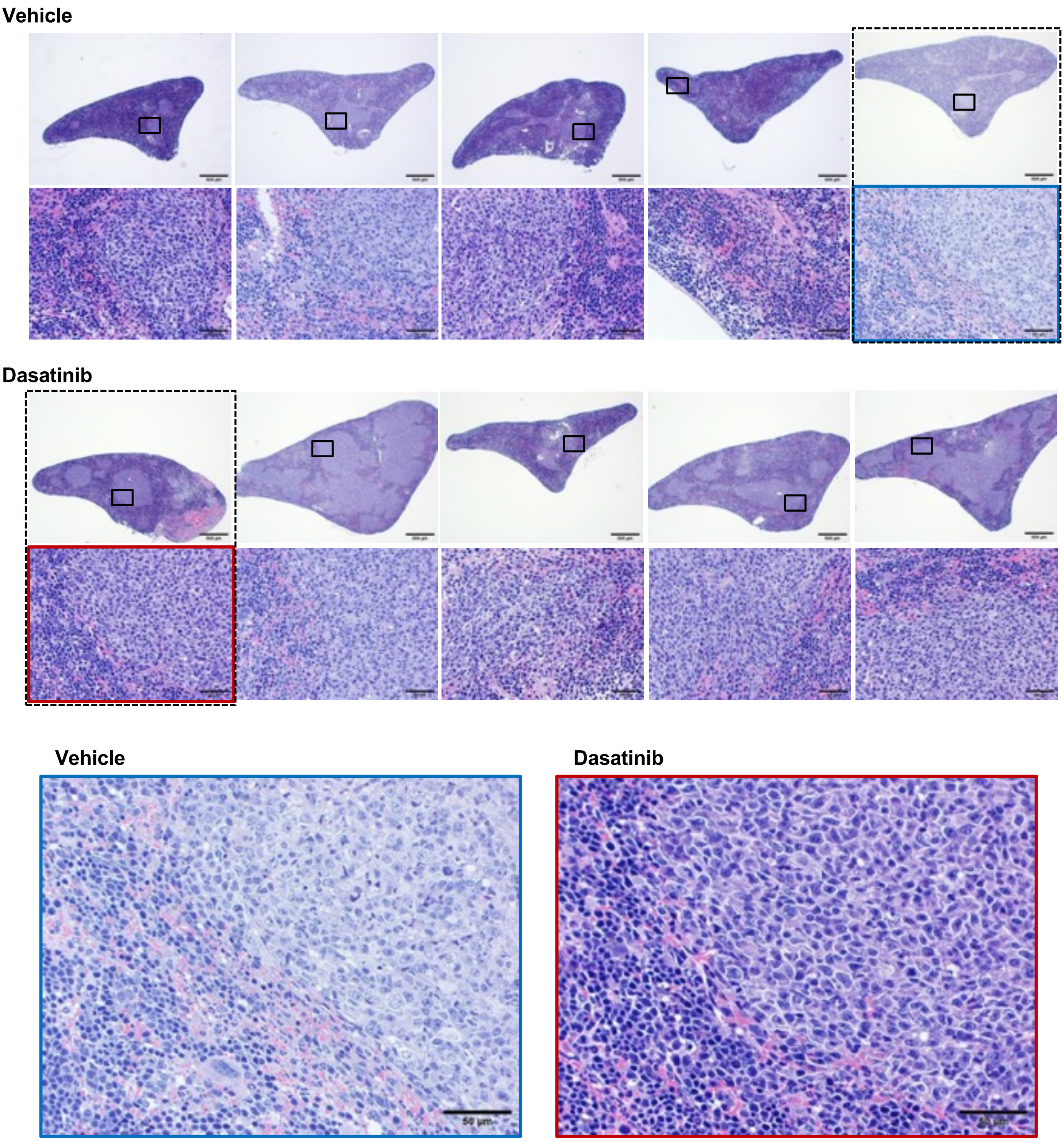

Figure S14. Infiltration of tumor cells into the spleen is exacerbated by dasatinib treatment.
Tissue sections of the spleens stained with hematoxylin and eosin were analyzed. The small images are from all of the mice treated with the vehicle or dasatinib (*n* = 5). Upper is low-powered images (scale bars: 500 µm), whereas lower is high-powered ones (scale bars: 50 µm), in each sample. On the bottom, representative high-powered images of vehicle- (blue-framed) and dasatinib- (red-framed) treated mice are enlarged for visibility. The images framed with a dashed line are also exhibited in Fig. 7D.

Figure S15.

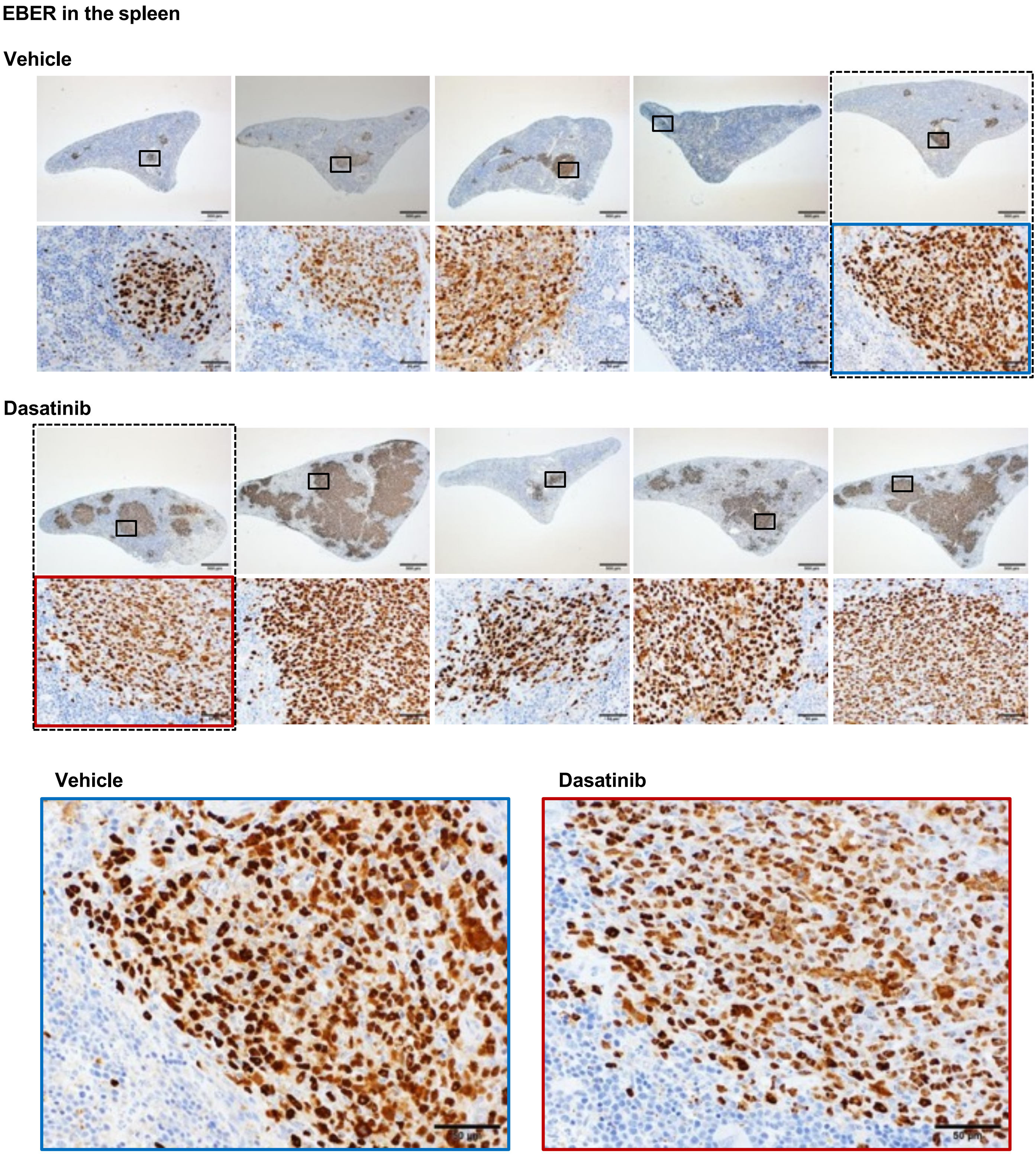

Figure S15. Infiltration of EBER⁺ tumor cells into the spleen is exacerbated by dasatinib treatment. Tissue sections of spleens stained with EBER ISH were analyzed. The small images are from all of the mice treated with the vehicle or dasatinib (*n* = 5). Upper is low-powered images (scale bars: 500 μm), whereas lower is high-powered ones (scale bars: 50 μm), in each sample. On the bottom, representative high-powered images of vehicle- (blue-framed) and dasatinib- (red-framed) treated mice are enlarged for visibility. The images framed with a dashed line are also exhibited in Fig. 7E.

Figure S16.

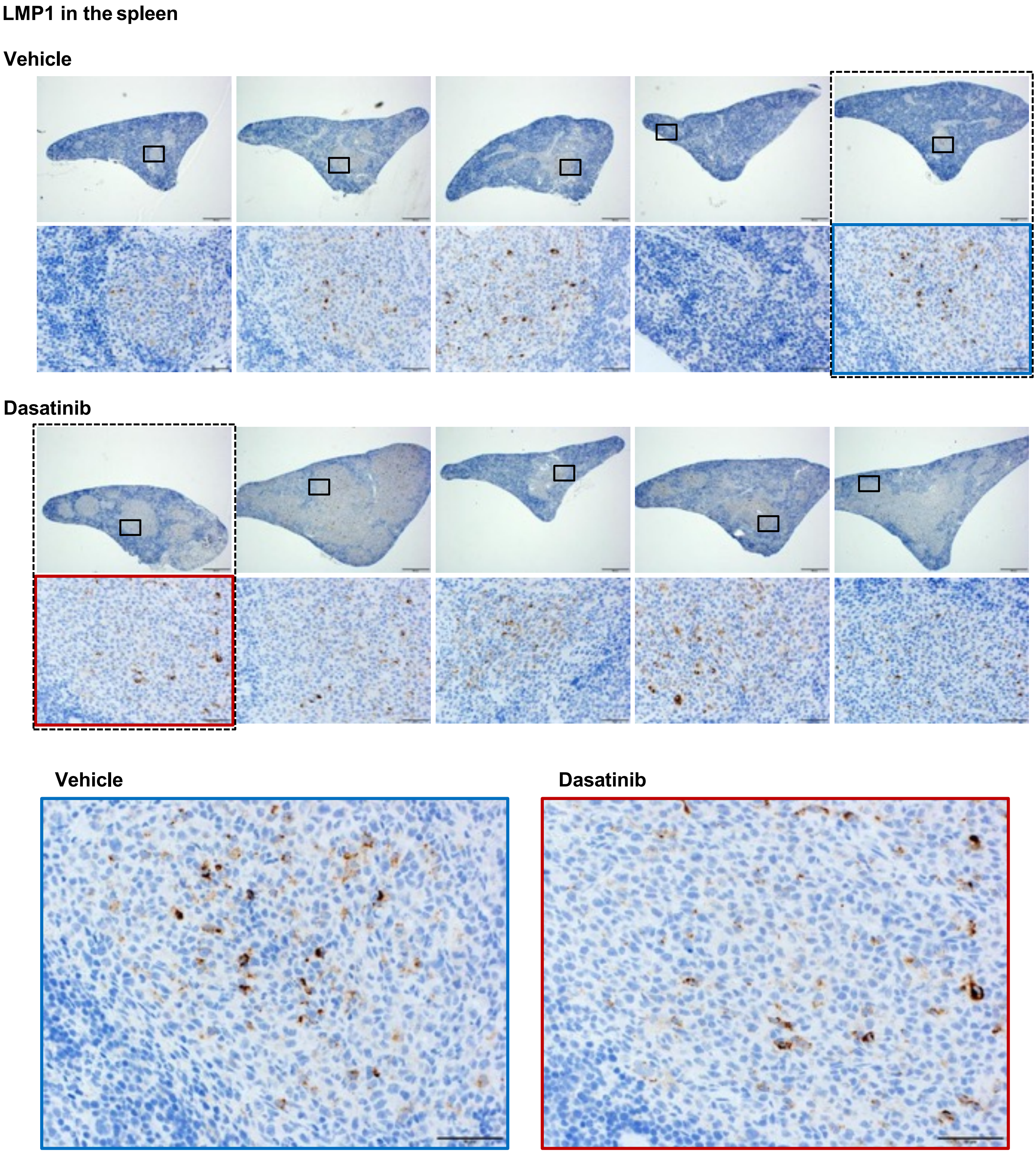

Figure S16. Infiltration of LMP1⁺ tumor cells into the spleen is exacerbated by dasatinib treatment. Tissue sections of spleens stained with anti-LMP1 Ab were analyzed. The small images are from all of the mice treated with the vehicle or dasatinib (*n* = 5). Upper is low-powered images (scale bars: 500 μm), whereas lower is high-powered ones (scale bars: 50 μm), in each sample. On the bottom, representative high-powered images of vehicle- (blue-framed) and dasatinib- (red-framed) treated mice are enlarged for visibility. The images framed with a dashed line are also exhibited in Fig. 7F.

Figure S17.

EBNA2 in the spleen

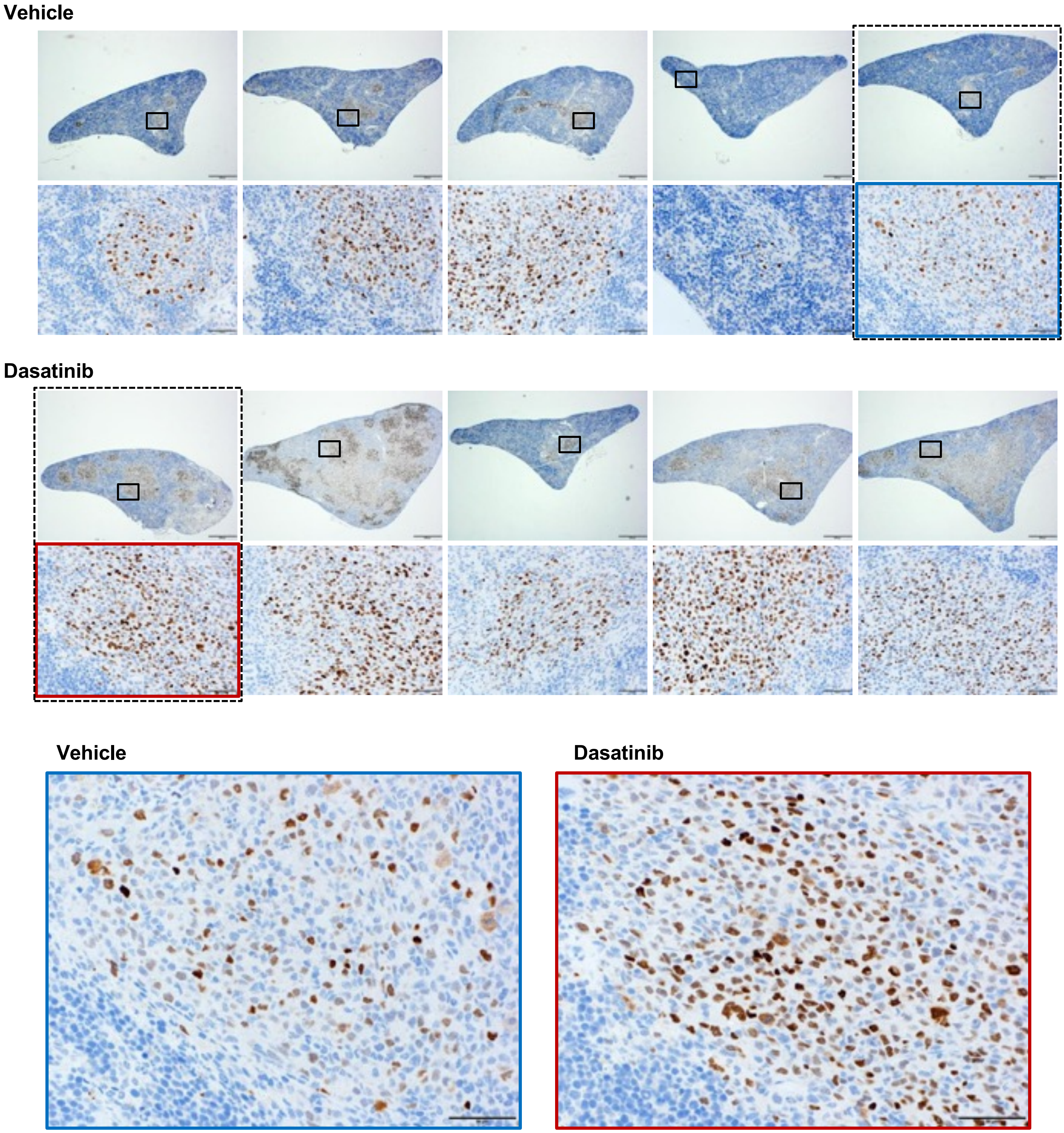

Figure S17. Infiltration of EBNA2⁺ tumor cells into the spleen is exacerbated by dasatinib treatment. Tissue sections of spleens stained with anti-EBNA2 Ab were analyzed. The small images are from all of the mice treated with the vehicle or dasatinib (*n* = 5). Upper is low-powered images (scale bars: 500 μm), whereas lower is high-powered ones (scale bars: 50 μm), in each sample. On the bottom, representative high-powered images of vehicle- (blue-framed) and dasatinib- (red-framed) treated mice are enlarged for visibility. The images framed with a dashed line are also exhibited in Fig. 7G.

Figure S18.

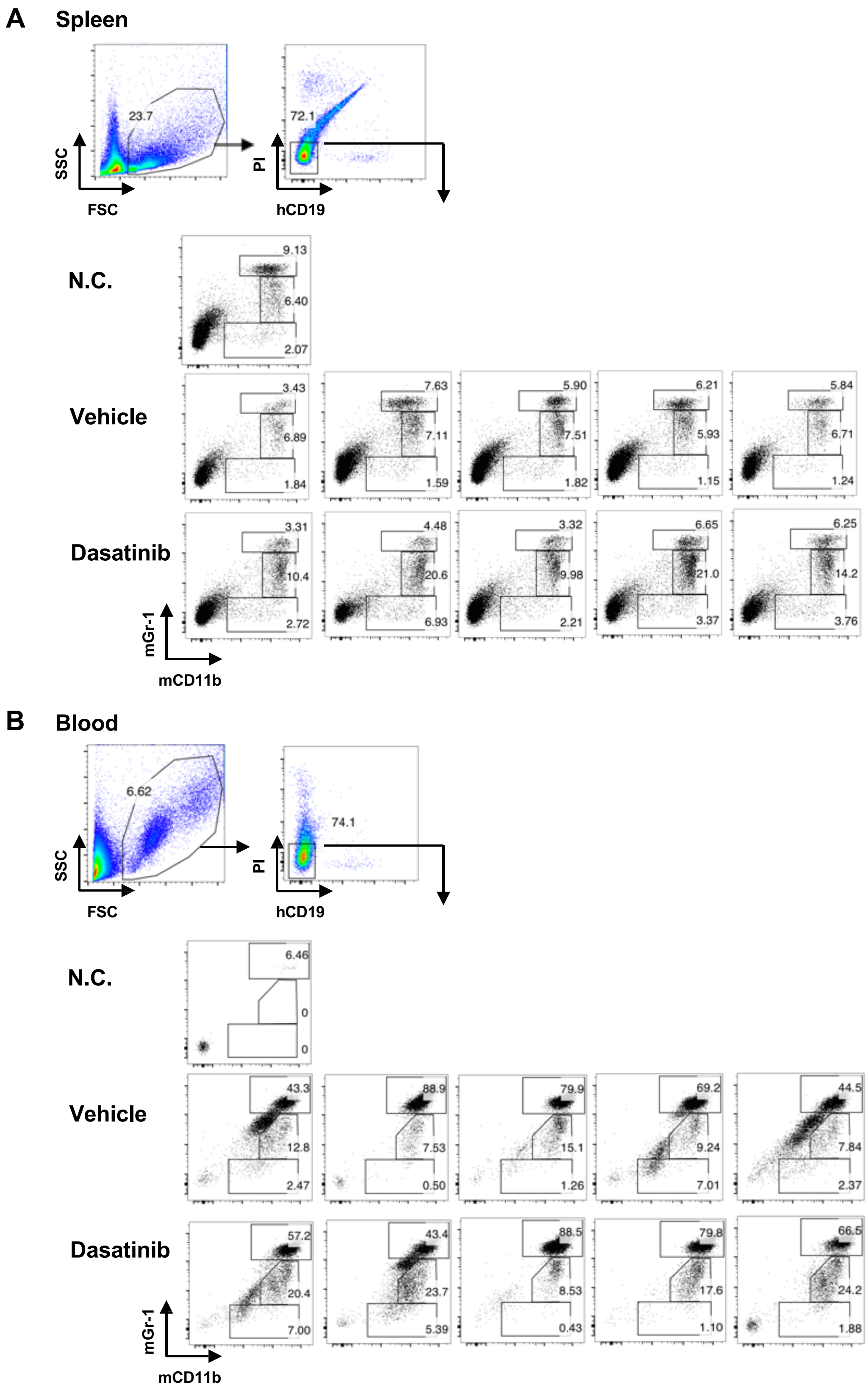

Figure S18. CD11b^{+}Gr-1$^{-/Low}$ cells increase in dasatinib-treated mice.
CD11b and Gr-1 staining on live non-tumor (hCD19^{-}PI^{-}) cells in the spleen (A) and the peripheral blood (B) of the vehicle- or dasatinib-treated mice were analyzed (*n* = 5). N.C. (negative control) indicates NOG mouse without tumor inoculation.

Figure S19.

Spleen

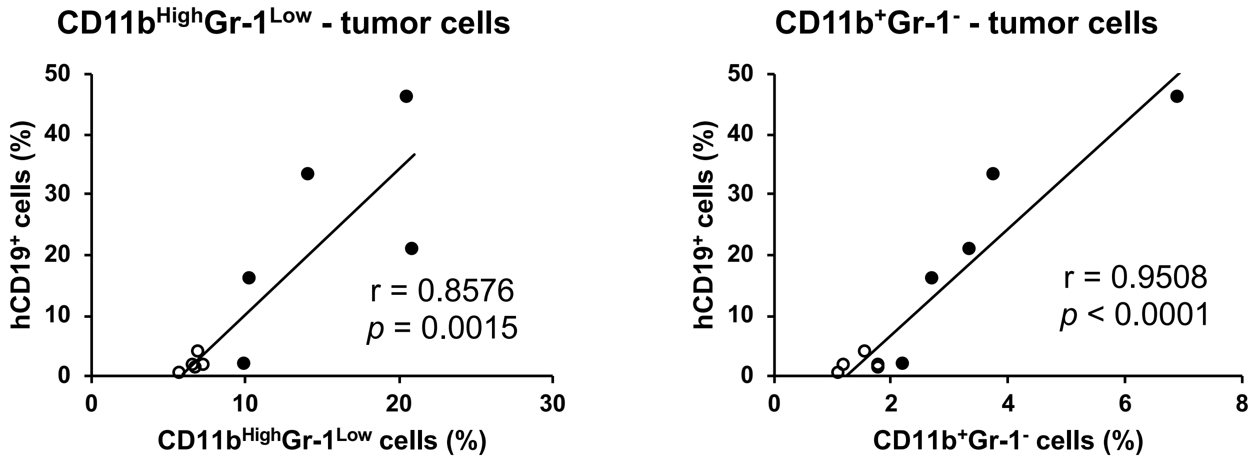

Blood

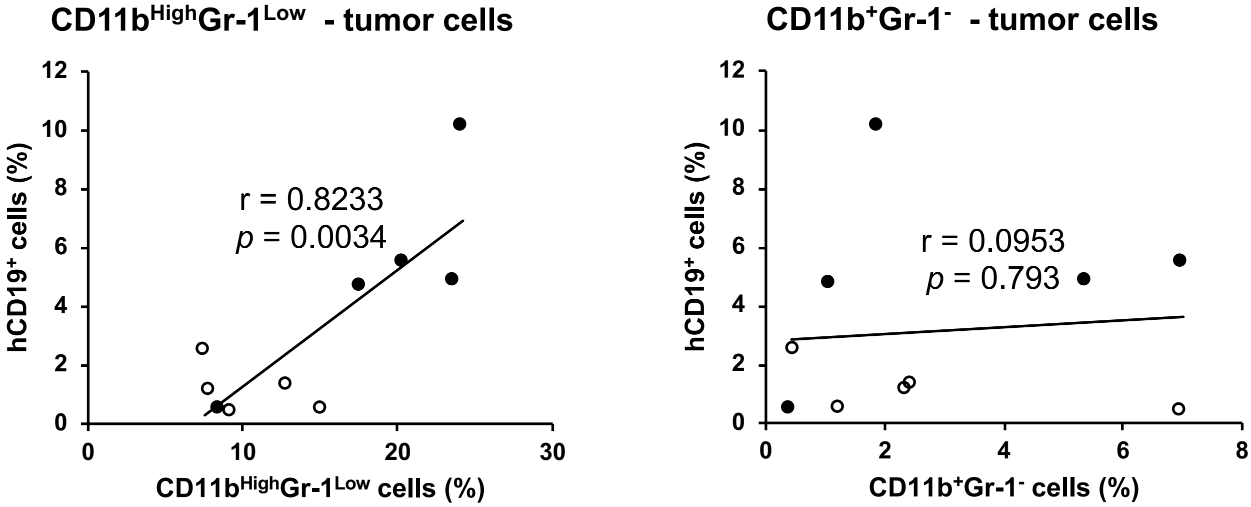

Figure S19. CD11b^{High}Gr-1^{Low} or CD11b^{+}Gr-1^{-} – tumor cells in spleen, and CD11b^{High}Gr-1^{Low} – tumor cells in blood showed positive correlation.
Correlation of indicated cells in spleen and blood of the vehicle (white plot) or dasatinib-treated (black plot) mice were analyzed (n = 5) using Pearson correlation. r (correlation coefficient) and p values are shown in each figure.
